# Bacterial Infections Following Splenectomy for Malignant and Nonmalignant Hematologic Diseases

**DOI:** 10.4084/MJHID.2015.057

**Published:** 2015-10-13

**Authors:** Giuseppe Leone, Eligio Pizzigallo

**Affiliations:** 1 Istituto di Ematologia, Università Cattolica del Sacro Cuore, Roma.; 2 Universita “G. d’Annunzio”, Chieti. (Italy)

## Abstract

Splenectomy, while often necessary in otherwise healthy patients after major trauma, finds its primary indication for patients with underlying malignant or nonmalignant hematologic diseases. Indications of splenectomy for hematologic diseases have been reducing in the last few years, due to improved diagnostic and therapeutic tools. In high-income countries, there is a clear decrease over calendar time in the incidence of all indication splenectomy except nonmalignant hematologic diseases. However, splenectomy, even if with different modalities including laparoscopic splenectomy and partial splenectomy, continue to be a current surgical practice both in nonmalignant hematologic diseases, such as Immune Thrombocytopenic Purpura (ITP), Autoimmune Hemolytic Anemia (AIHA), Congenital Hemolytic Anemia such as Spherocytosis, Sickle Cell Anemia and Thalassemia and Malignant Hematological Disease, such as lymphoma. Today millions of people in the world are splenectomized. Splenectomy, independently of its cause, induces an early and late increase in the incidence of venous thromboembolism and infections. Infections remain the most dangerous complication of splenectomy. After splenectomy, the levels of antibody are preserved but there is a loss of memory B cells against pneumococcus and tetanus, and the loss of marginal zone monocytes deputed to immunological defense from capsulated bacteria. Commonly, the infections strictly correlated to the absence of the spleen or a decreased or absent splenic function are due to encapsulated bacteria that are the most virulent pathogens in this set of patients. Vaccination with polysaccharide and conjugate vaccines again *Streptococcus pneumoniae, Haemophilus influenzae,* and *Neisseria meningitidis* should be performed before the splenectomy. This practice reduces but does not eliminate the occurrence of overwhelming infections due to capsulated bacteria. At present, most of infections found in splenectomized patients are due to Gram-negative (G-) bacteria. The underlying disease is the most important factor in determining the frequency and severity of infections. So, splenectomy for malignant diseases has the major risk of infections.

## Introduction

*A 73-year-old man, affected by splenic lymphoma with massive splenomegaly underwent to elective splenectomy at seventy. He has been suffering from splenic lymphoma for ten years and on therapy with Chlorambucil and Rituximab; the indication for splenectomy was an enormous spleen resistant to chemo - immunotherapy and a mild thrombocytopenia.*^1^ *He received the 23-valent pneumococcal polysaccharide vaccine (PNEUMOVAX 23) after surgery that he repeated two years later. Three years after his surgery, he calls his primary care doctor because he has fever and cephalgia. What is the appropriate management? Patients splenectomized for a hematologic disease are at major risk than subjects splenectomized for trauma? Which prophylactic measures and which therapy are indicated?*

Splenectomy, while often necessary in otherwise healthy patients after major trauma,^2^–^3^ find its primary indication for patients with an underlying malignant or nonmalignant hematologic diseases (Table 1).^2^–^12^ Rarely spleen rupture can occur spontaneously, more frequently in a pathological spleen for infectious or/and hematologic diseases^13^–^17^ and in patients on anticoagulation.^18^ People without risk factors or previously diagnosed disease can, even if rarely,^19^ undergo to splenic rupture for minor trauma o if treated with high dose of growth factors for stem cell harvest.^19^ Furthermore, functional asplenia, due to auto infarction, frequently develops in subjects with sickle cell anemia.^16^ Also, hyposplenism states are common in patients with chronic graft-versus-host disease after stem-cell transplantation, severe celiac disease, and untreated human immunodeficiency virus infection.^20^

**Figure 1:**
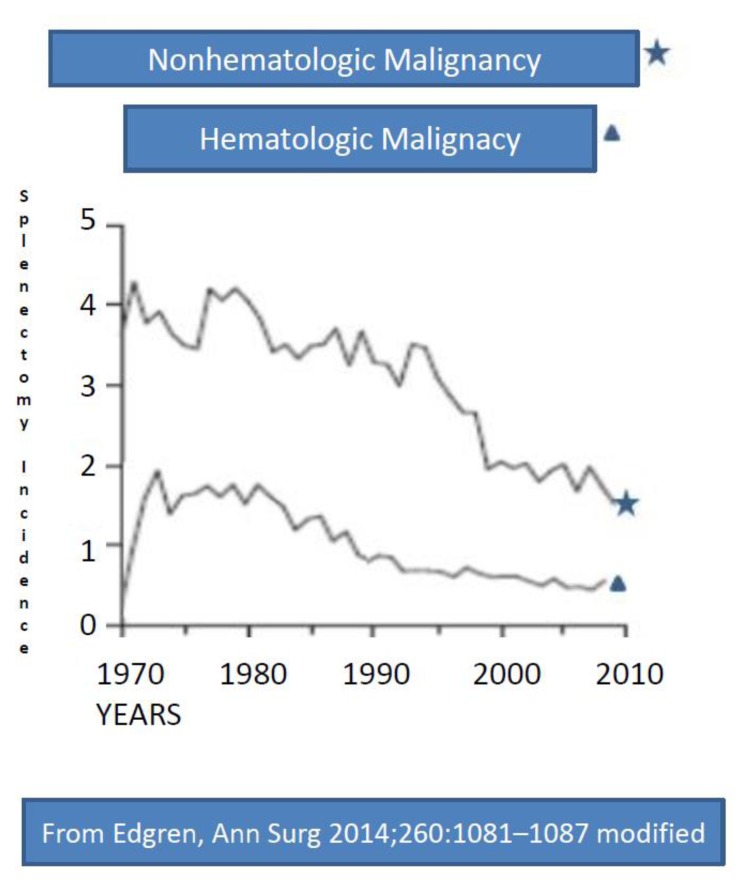
Number of splenectomy throughout the recent years.

**Figure 2:**
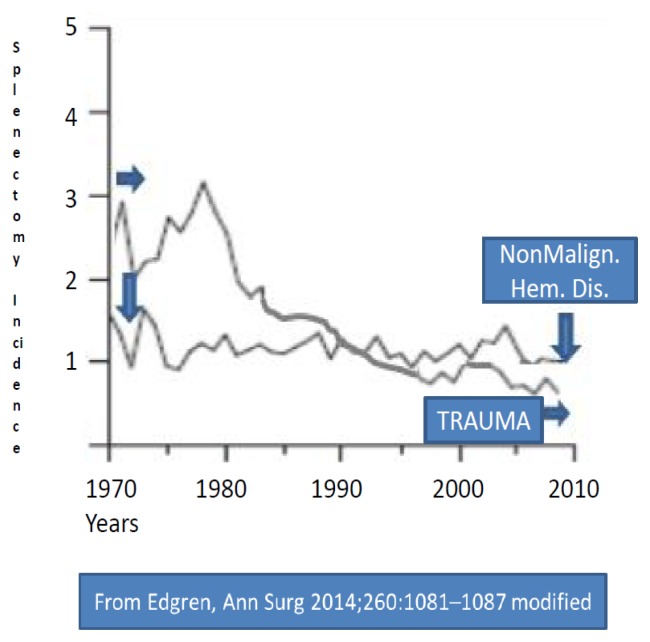
Number of splenectomy throughout the recent years.

Indications of splenectomy for hematologic diseases have been reducing in the last few years, due to improved diagnostic and therapeutic tools (Figure 1,2).^8^ Reduction of splenectomy is even more evident after trauma since splenic preservation has become a well-reported and accepted principle.^8^,^21^ Splenectomy for cancer staging is infrequently performed,^2^ and no longer requested for Hodgkin Disease (HD) staging, as in the past,.^22^ The introduction of rituximab has reduced the necessity of splenectomy for some lymphoproliferative diseases,^1^ hemolytic anemia and ITP.^12^ At present the splenectomy sometimes can also be avoid by treating resistant ITP patients with thrombopoietin-receptor agonists.^12^,^23^ All these data infer that the indications for splenectomy continue to evolve, with a progressive reduction, more evident after trauma and in malignant hematologic diseases.

However, splenectomy, even if with different modalities including laparoscopic splenectomy and partial splenectomy,^24^,^25^ continue to be a current surgical practice. Approximately 25,000 surgical splenectomies are performed annually in the United States;^26^ and, the total number of asplenic persons in the United States is currently estimated at 1 million, including 70,000 to 100,000 persons with sickle cell disease.^27^ Data in the other countries are not available. In clinical practice splenectomy is performed worldwide for different reasons according to the prevalence of different pathologies, circumstances and availability of drugs, found in every country (Table 1).

In high-income countries, like USA, Australia, Europe at present, the proportion of splenectomy secondary to trauma represents the 15–30% of all cases (Table 1).^2^,^5^,^7^,^8^ This percentage is lowering, Some years ago (2001) Bisharat reported a percentage of splenectomy due to trauma in 50% of adults and 30% of children.^4^ However, in high-income countries there is a clear decrease over calendar time in the incidence of splenectomy for all indications except nonmalignant hematologic diseases (Figure 1 and 2).^8^

In the low-income country and war period, the proportion of trauma splenectomy could be higher.

Khamechian^28^ report in Iran a percentage of 75% of trauma splenectomy and Deodhar report similar results in India (Table 1).^29^

Among the non-traumatic splenectomy hematologic indications are prevalent but differ in the various countries and the different series. In Europe and USA the prevalent indications of splenectomy are represented by the lymphoproliferative diseases (more frequently in the hospitals with prevalent oncological patients, such as the Memorial Sloan-Kettering Cancer Center, New York, USA^9^ and by the ITP (more frequently in the General Hospitals, as reported by two important series of American College of Surgeons^10^ and by the Swedish Study.^8^ In Asia and in Africa hemoglobin disorders are the prevalent indication for splenectomy (Table 2).^30^,^31^

Programmed splenectomy is made more and more frequently by laparoscopy, which is mostly utilized for benign spleen-related diseases.^24^,^32^ However at variance with European and USA experience, in the Chinese and Asian series, portal hypertension and hypersplenism, secondary to cirrhosis is an important cause of splenectomy.^32^,^33^ The study of Wang et Coll.^32^ retrospectively reviewed 302 consecutive patients who underwent laparoscopic splenectomy. 65% of patients had a benign spleen-related disease, 14% a malignant spleen-related disease and 21% portal hypertension. In a similar Italian series portal hypertension does not appear as cause of splenectomy.^24^

## Complications of Splenectomy

Splenectomy, independently of its cause, induces an early and late increase in the incidence of venous thromboembolism and infections.^4^–^12^,^34^,^35^ The previous pathology influences the incidence of both complications.^35^,^36^ Therefore, the comparison should be made with a matched indication cohort.^5^ However, in any case, infection remains the most noxious complication of splenectomy.^4^,^8^,^34^,^35^

### Infections

Commonly, the infections strictly correlated to the absence of the spleen or a decreased or absent splenic function are due to encapsulated bacteria that are the most virulent pathogens in this set of patients.^2^–^8^ They can produce a serious fulminant illness, called overwhelming post-splenectomy infection (OPSI), that carries a high mortality rate.^4^,^8^,^36^,^37^,^38^ However, in the years, the bacterial pattern of splenectomy sepsis have been changing. The most important capsulated pathogen is *Streptococcus pneumoniae* (*Str. Pneumoniae*), but *Haemophilus influenza* (*H.Influenzae*) and *Neisseria meningitidis* (*N*. *meningitidis*) are also significant. In a study of 1991,^36^ reporting 349 episodes of sepsis in patients with asplenia, 57% of infections and 59% of deaths were caused by *Str. pneumoniae*. Furthermore, 6% of infections were caused by *H. influenza*e, with a mortality rate of 32%; *N. meningitidis* was the organism in 3.7% of cases in the same study.^38^ Today, after the introduction of vaccination, and oral penicillin antibiotics, patients submitted to splenectomy can suffer from disparate strains of bacterial infection, which are not strictly correlated with the splenic function. In fact, particularly in the post-intervention phase, the type of bacteria isolated in the blood is not so different from those found in other abdominal interventions. So, gram- bacteria are prevalent (51% in the Australian report).^6^,^8^,^38^ At present in vaccinated patients, the rate of sepsis by pneumococcus is very low. In fact, encapsulated bacteria, such as *pneumococcus*, *meningococcus*, and *H. influenzae*, were rarely encountered in Australian and Danish cohort Series,^6^,^11^,^38^ in whom vaccination was routinely adopted.

However, the infection from capsulated bacteria continue to be important because vaccination does not cover all bacterial strains and assumes a particular virulence in patients with absent or reduced splenic function. OPSI, also today, has a mortality of 30–60%.^37^ Sepses by uncommon bacteria^39^–^40^ as well by protozoa infections such as malaria and babesiosis are also known to affect asplenic patients.^41^–^43^

Why the asplenic patients are so sensitive to encapsulated organism?

The spleen was once considered unnecessary for life; however, it clearly serves extremely important hematologic and immunologic functions. Spleen function consists of several aspects, according to the three anatomical splenic subunits: (a) the white pulp, containing B-cell follicles, (b) the marginal zone (MZ), containing specialized macrophages and memory B-cells, and (c) the red pulp, where erythrocytes are filtered from the circulation by entrapment in the splenic cords and subsequent phagocytosis, as well as by retention through receptor ligand interaction.^44^

The white pulp contains a large mass of lymphoid tissue and serves a vital role in the recognition of antigens and production of antibodies. The red pulp of the spleen consists of a tight meshwork of sinusoids, the cords of Billroth, which primarily serve hematologic functions, especially filtration of the blood. The milieu of the red pulp is relatively acidic and hypoglycemic. Therefore aged or damaged red cells not able to tolerate this harsh environment are ultimately removed by splenic macrophages.^44^ Particulate matter is also removed from red cells as they pass through the splenic sinusoids, and so “polished” or “conditioned” red cells, free of surface imperfections, come back to the bloodstream. The red pulp also acts as a reservoir for approximately one-third of the total platelet mass and a smaller proportion of granulocytes.

Both lymphocytes and monocytes present in the spleen are important to assure a complete immunological defense. MZ B cells have a unique ability to produce natural antibodies and can initiate T-cell independent immune responses to infections or vaccination with capsular polysaccharide antigens. In fact, the human immunoglobulin M memory B cells controlling *Str. pneumoniae* infections are generated in the spleen.^45^–^54^ After splenectomy, the levels of antibody are preserved but there is a loss of memory B cells against pneumococcus and tetanus.^51^ The fundamental rule of splenic monocytes in the immunological defense from capsulated bacteria should be always taken in consideration.^54^–^55^

The most conspicuous macrophage populations of the spleen are located in the marginal zone and adorned with unique sets of pattern recognition receptors. The MZ is a strategically positioned in the bloodstream and contains both macrophages and memory B cell.^46^ The macrophage subsets present in the spleen marginal zone show various pathogen receptors on in the recognition and elimination of certain pathogens, in particular, encapsulated bacteria.^55^,^56^ It is noteworthy that complement defects induce streptococcal and meningococcal infections very similar to that found in splenectomized subjects.^57^ Complement system, such as C1q and C3, and macrophages in the splenic marginal zone (sMZ) play pivotal roles in the efficient uptake and processing of circulating apoptotic cells. SIGN-R1, a C-type lectin that is highly expressed in a subpopulation of MZ Macrophages, regulates the complement fixation pathway by interacting with C1q, to fight blood-borne *Streptococcus pneumoniae*.^57^–^59^. SIGN-R1+ macrophages are critical for the uptake of circulating apoptotic cells in the MZ and are essential for *Str. pneumoniae* clearance.^55^–^57^

In conclusion, the specific role in the removal of encapsulated bacteria is related to marginal zone macrophages, which can detect and capture encapsulated bacteria.^54^–^57^ In addition, marginal zone cells respond to capsule polysaccharide antigens by differentiating into IgM-producing memory B cells or antigen presenting cell.^56^–^57^

At present splenectomy is performed both in subjects with and without a previous pathology. Therefore, we firstly treat the infections of healthy people splenectomized as a consequence of trauma, considering them as a control group. Accordingly the literature^2^–^12^ we make an important distinction between the early post intervention infections and the late infections. Afterward, we consider pathology by pathology the different hematologic groups requiring splenectomy. In fact, the previous pathology does influence the rate, the type and the severity of the early as well the late infections.

## Early Infections

Infections related to splenectomy can occur early in direct association with intervention (post-operative infectious complications) and late in connection only with the reduced immunological defense induced by splenectomy. Infective complications account for most of the perioperative morbidity and include lower respiratory tract infections, intra-abdominal collections, wound infection and non-specific infections requiring antibiotics.^5^–^10^ The Danish series^5^ reports 3812 persons who underwent splenectomy from 1996 to 2005. The maximum relative risk of infection and death was within the first 90 days of intervention, attaining a RR of about 20 fold higher in all indication groups than in the general population comparisons, whereas odds ratios in comparison with appendicectomized patients ranged from 1.0 to 12.7.

**Figure 3:**
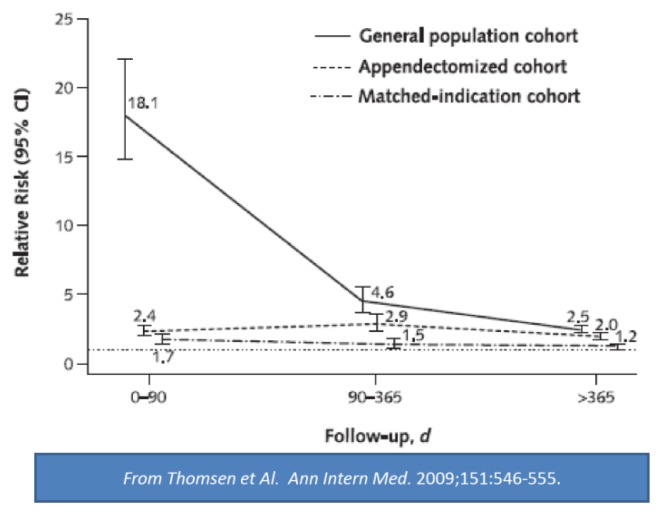
Relative risk of infections after splenectomy with different matchings.

The distribution of microbial agents was similar between groups. Of note, encapsulated bacteria, such as pneumococci, meningococci, and *H. influenzae*, were rarely encountered in the splenectomized cohort, recently reported in the west countries.^5^,^11^ Similarly the adjusted relative risk (RR) and 95% confidence interval (CI) of death among splenectomized patients by indication, compared to the general population of Denmark, was the highest in the first 90 days, attaining a RR of 33-fold. However, although splenectomized patients have a high risk for infection, this risk is different in the various subgroups, and some degree seems due to underlying conditions and not to splenectomy alone. (Figure 3) The risk of death within the first 90 days ranges from 2,5% in patients splenectomized for ITP to 10% in patients with hemopoietic cancer or trauma.^5^ Older age can also be an important factor in increasing infection morbidity and mortality in the post-intervention period in elective splenectomy of hematologic patients.^6^,^12^

Heuer^59^ report the Germany experience of 1,630 patients with a splenic injury, whose, 758 patients undergoing splenectomy compared with 872 non-splenectomized patients. 96 (18.3%) of the patients with splenectomy and 102 (18.5%) without splenectomy had an apparent infection after the operation. Additionally, there was no difference in mortality (24.8% versus 22.2%) in both groups. Patients with minor trauma take advantage from conservative treatment, at contrary patients with major trauma take advantage from splenectomy. It is important to note that the perioperative sepsis rate was the same in both groups.^59^

Bickenbach et al.^9^ report in 2013 the MD Anderson experience of 381 patients, who underwent splenectomy for diagnosis or treatment of hematological diseases. Overall 136 patients (35.7 per cent) experienced complications. Independent predictors of any morbidity on multivariable analysis were age more than 65 years, KPS score 60 or less, and hemoglobin level 9 g/dl or lower. The complications in this series were mainly infectious (41,9 %), and the majority of the deaths were directly related to infections. The microorganisms involved in the infections were not cited.

Barmparas et al.^34^ compared 2 groups of patients submitted to abdominal surgery including or not splenectomy. In a series of 493 patients submitted to abdominal surgery, 33 underwent to splenectomy too, the two groups were well balanced for age. Patients undergoing splenectomy were more likely to have sustained a traumatic injury (30% vs. 7%, p < 0.01). After adjustment, splenectomy was associated with increased risk for infectious complications (49% vs. 29%, Adjusted Odds Ratio (AOR) [95% CI]: 2.7 [1.3, 5.6], p <0.01), including intra-abdominal abscess (9% vs. 3%, AOR [95% CI]: 4.3 [1.1, 16.2], p < 0.03). On a subgroup analysis, there were no differences between traumatic and elective splenectomy with regards to overall infectious complications (50% vs. 46%, p = 0.84), although, abdominal abscess developed only in those who had an elective splenectomy (0% vs. 12%, p =0.55). The authors concluded that splenectomy increased the risk for postoperative infectious complications. In fact, even when the intra-abdominal diseases were eliminated, splenectomy increased the risk for early overall infectious complications and postoperative intraperitoneal abscess. However the increase the post-intervention infections could not induce a significant increase in early mortality.^58^, ^59^ In adult patients the early mortality raises with age^6^ particularly in patients with hematologic neoplasms.^9^

The laparoscopic approach to splenectomy is clearly superior to standard laparotomy in terms of postoperative complications, including infections,^60^ although the rate of OPSI remains similar in early as well in late phase.^37^,^60^ In fact, most of these early post-splenectomy bacteremia was caused by Enterobacteriaceae, *Pseudomonas spp* and *Staphylococcus spp*, and occurred mostly in patients with gastrointestinal malignancies while *Str. pneumoniae* caused only a few.^6^,^7^,^8^

In conclusion, it seems that the splenectomy does not significantly influence the type of the early infection that is mostly related to surgical trauma. Laparoscopic approach reducing surgical trauma reduces infections rate and early mortality.

## Late Infections

### Patients Splenectomized After Trauma Without A Previous Pathology

Patients without a previous pathology are splenectomized because of trauma, and rarely for spontaneous rupture or after G-CSF. The difference in the incidence of bacterial infections could depend on the age at splenectomy. The sepsis incidence and mortality is higher in children than in adult,^4^,^5^,^8^ the recent Swedish experience^8^ confirm the previous data of Bisharat et al. .^4^ The incidence of Sepsis, expressed as standardized incidence ratios (SIR), varied with age and follow-up, with the highest SIRs among children. When restricting to those who were splenectomized at the age of 0 to 12 years, the SIR was higher: 6.1 (95% CI, 3.3–10), in respect of total population, SIR of 3.1 (95% CI, 2.1–4.3).

However in the adults the older age is a negative factor both in term of morbidity and mortality.^5^

The Swedish experience^8^ takes into consideration only the splenectomized patients after 180 days from intervention, (20,132 patients), excluding them who either died or were censored within 180 days of first discharge.^8^ The cumulative incidence of first hospitalization for or death from sepsis varied both by indication and calendar year of splenectomy. The overall 30-day mortality after a hospitalization for sepsis was 17% (372 deaths after 2243 hospitalizations) and ranged from 13% for patients splenectomized for trauma to 22% for those splenectomized for a hematologic malignancy. In all, there were 2243 hospitalizations for sepsis, corresponding to an overall nearly six-fold increased risk of sepsis (SIR 5.7; 95% CI, 5.6–6.0). The risk of a new hospitalization for sepsis varied by indication, with the lowest risk among the trauma patients (SIR 3.4; 95% CI, 3.0–3.8) and highest among the hematologic malignancy patients (SIR 18; 95% CI, 16–19). SIRs varied with age and follow-up, with the highest SIRs among young patients, and in the earliest follow-up periods after the splenectomy. The incidence of sepsis was higher in the first 2 years, but it remain higher also after ten years.^7^,^10^

Kristnsson and others^10^ have reported infectious and thrombo-hemorrhagic complications in American veterans, cancer free, submitted to splenectomy for different reasons. No differences were found in term of infections between patients splenectomized for trauma and those splenectomized for hematological nonmalignant diseases. In the late follow-up infections from capsulated bacteria in patients splenectomized after trauma become prevalent in the veteran American series^9^ but not in the Danish series,^5^ in which the percentage of *Str. pneumoniae* infection is only 4%.

In the American series splenectomized patients had a significantly increased risk of pneumococcal pneumonia (RR=2,06, meningitis RR=2,44 and septicemia 3.44), however, the risk of death is particularly increased only from septicemia and meningitis. In Denmark, pneumococcal vaccination is recommended within 2 weeks before elective splenectomy, or a soon as possible and within less than 2 weeks after emergent splenectomy, but no vaccination for *H. influenza*e is recommended. Neither of two studies would give sufficient data about the vaccination, even if both stressed the importance of vaccination. In any case from epidemiological data, it is evident that there is a reduction of *Str. pneumoniae* infections since the vaccination is beginning to be a routine procedure in most countries.^6^,^7^,^38^

### Patients Splenectomized For Hematologic Diseases

#### Nonmalignant Diseases

Splenectomy also represents at present a key treatment option for the treatment of many benign hematological diseases, including immune thrombocytopenia (ITP), Auto Immune Hemolytic Anemia (AIHA) and hereditary disorders associated with ongoing hemolysis (Spherocytosis, Thalassemia major and intermedia, Sickle cell anemia).^11^,^12^ In fact, the number of patients splenectomized for hematological non-malignant diseases remains stable and at present represent the most frequent indication for splenectomy in high-income countries.^8^ However, among the hematological non-malignant diseases with a sound indication to splenectomy, we must distinguish the acquired diseases, ITP, AIHA in which the autoimmunity play a fundamental role, and the congenital forms, such as Spherocytosis and Hemoglobin disorders.

##### Immune Thrombocytopenic Purpura (ITP)

Although new drugs such as Rituximab and Thrombopoietin analogs have been introduced in the treatment of ITP resistant to steroids, the splenectomy remains the gold standard for the therapy of resistant patients.^12^ At present ITP represent in many western series the larger indication to splenectomy.^5^,^8^,^10^ Splenectomy remains the only treatment that appears to have a long lasting effect in patients with ITP.^12^,^61^–^63^ Response rates are around 70% in children with chronic ITP and 60 % in adults. The guidelines show considerable differences in recommendations for splenectomy.^12^,^61^ The more recent ASH guidelines^61^ recommend delaying surgery to after 12 months vs. six months as recommended in the past. Infections remain the major contraindication to splenectomy in ITP, particularly in children.^12^,^61^, However, it is important to consider that also the immunosuppressive agent increase the incidence of infection. Therefore, the comparison should be made between resistant patients treated with the splenectomy or those treated with immunosuppressive agents (Figure 3).^65^,^67^,^68^

In a large series, Boyle et Al.^35^ report a cohort of 9976 patients with ITP; all patients were 18 years of age or older and had a diagnosis of ITP, as the main disease, from January 1990 to November 2009. 1762 of them underwent splenectomy.

The cumulative incidence of sepsis was 11.1% among the ITP patients who underwent splenectomy and 10.1% among the patients who did not. Splenectomy was associated with a higher adjusted risk of sepsis, both early (HR 3.3 [CI, 2.4–4.6]) and late (HR 1.6 or 3.1, depending on comorbidities). He concludes that ITP patients post-splenectomy are at increased risk for abdominal venous thromboembolism (AbVTE), venous thromboembolism, (VTE), and sepsis.

Sepsis developed in 1016 cases: 191 splenectomized cases (cumulative incidence 11.1%) and 825 nonsplenectomized cases (cumulative incidence 10.1%), with a median follow-up of 56 months. The cumulative incidence of early sepsis after splenectomy (<90 days) was 2.6% and of late sepsis (>90 days) was 8.8%. Among the splenectomy cases, the median time from splenectomy to hospitalization with sepsis was 35.5 months (range, 0–219). In the multivariable model for sepsis, splenectomy was a significant predictor of both early and late sepsis, with a more than threefold higher hazard ratio (HR) for early sepsis (HR 3.3 [CI, 2.4–4.6]). For late sepsis, there was an interaction between splenectomy and number of comorbidities. Cases with none or one comorbidity had an HR of 1.6 (CI, 1.3–2.0), and for cases with 2 or more comorbidities, the HR was 3.1 (CI, 2.2–4.4). There was also an interaction between age and number of comorbidities. In addition to splenectomy, age >60 years, the presence of comorbidities, the male sex, and the African ethnicity, were also significant predictors of sepsis.^35^ In a retrospective analyze, Vianelli et al.^63^ reported 233 ITP adult patients, who underwent splenectomy between 1959 and 2001 in 6 European hematologic institutions and who have now a minimum follow-up of ten years from surgery. Of the 233 patients, 180 (77%) achieved a complete response and 26 (11%) response. Sixty-eight of 206 (33%) responsive patients relapsed, mostly (75%) within four years from the first response. In 92 patients (39.5%), further treatment was required after splenectomy that was effective in 76 cases (83%). In 138 patients (59%), the response was maintained free of any treatment at last contact. Overall, 73 patients (31%) experienced at least one infectious complication, for a total of 159 events, most often pneumonia (40%). Forty-three of these patients (59%) had received prophylactic vaccinations. Median time from splenectomy to the first infection was 35 months (range 0–355). Infectious complications were significantly more frequent in refractory patients compared to stable responders (P=0.004) but were comparable (P>0.05) in vaccinated and non-vaccinated patients. Two fatal infectious episodes (sepsis and intestinal infection) occurred, after 176 and 318 months from splenectomy. Both patients were stable responders to splenectomy and were 78 and 80 years old.

Today to avoid splenectomy and the consequent major infection rate, alternative treatments are performed in patient with resistant ITP. In the last few years, rituximab has been indicated as the first line treatment of resistant ITP patients.^64^ However, Rituximab is not free of side effects.^65^ Recently a study^65^ assessed the safety in 248 adult patients with immune thrombocytopenia (ITP) treated with rituximab. In total, 173 patients received four infusions of 375 mg/m2 and 72 received 2 fixed 1-g infusions two weeks apart. The authors observed 11 cases of infection in 7 patients (3%; 95% CI, 1–6) corresponding to an incidence of 2.3 infections/100 patient years (95% CI, 1.2–4.1). Infections occurred 2 to 18 months after the first rituximab infusion. Eight cases are recovered, but three patients died of infection 12 to 14 months after the first rituximab infusion. These patients were older than 70 years, 2 had severe comorbidities (diabetes and peritoneal carcinosis), and they had received prolonged treatment with corticosteroids for refractory ITP. Theses series of patients was not vaccinated, and the cause of infections was due to capsulated bacteria in two cases and both recovered.

At present the French guidelines^67^,^68^ recommend the vaccinations against *Streptococcus pneumoniae*, *Haemophilus influenzae* b (Hib) and *Neisseria meningitidis* not only before splenectomy but also before rituximab in patients aged less than 65. However, also in France this vaccination was made in a small proportion of patients (32.4%, 18.9%, and 3.8%. respectively). Furthermore, it is worth of noting that advanced age and comorbidities are the major risk factors for infections.^69^

The splenectomy was considered particularly dangerous in children in the past, the risk of fatal post-splenectomy sepsis was found to be severe especially in children less than five years and during the first year the following splenectomy.^4^,^5^,^7^ The mortality rate of children is higher than of adults.^8^ The mortality risk is estimated to be of 3% in children.^66^ The infectious risk in children and adults splenectomized for ITP is similar to that of children splenectomized after trauma.^4^,^5^,^8^,^11^ The increased risk compared with the general population persists for life.^12^ It is evident vaccinations does not eliminate post-splenectomy sepsis. However even if there are limited comparative data on the efficacy of vaccinations against encapsulated bacteria, is evident by the recent epidemiological data that vaccination reduces the incidence of infections by capsulated bacteria.^70^–^73^ In fact, in more recent publications,^70^–^72^ when vaccinations for pneumococcus and meningococcus are more and more becoming frequent, the capsulated infections are becoming rarer. The Intercontinental Childhood ITP Study (ICIS) Group Registry^71^ reported 134 children splenectomized in 57 institutions of 25 countries over a period of 225.2 patient-years. Of the 134 children in the ICIS Splenectomy Registry, 65 underwent a laparoscopic procedure, and perioperative bleeding occurred in eight patients, three of whom had laparoscopic splenectomy; four patients received packed red blood cells, postoperative fever was reported in 9.7% without signs of infection. This group signaled seven episode of sepsis (0.031sepsis episodes per patient-year), without a fatal outcome. In this study 21 patients were not submitted to vaccination, however of the seven episodes of sepsis only one was found in not vaccinated patients. The bacteria isolated was not reported in this paper but in the discussion was affirmed that “Sepsis caused by encapsulated bacteria was rarely encountered in patients on this Registry” independently of vaccination.

Similarly, Aladjidi et al.^72^ conducted retrospective analysis in 16 French departments involving 78 children with ITP and splenectomy.). Sixty-two children had chronic ITP of more than 12 months; laparoscopic splenectomy was utilized in 81% of children. Four patients experienced postoperative complications: two severe hemorrhages, one mesenteric thrombosis, and one pulmonary atelectasis. All four patients with complications had preparation by at least one platelet-enhancing treatment. Severe infections were not reported.

The choice of splenectomy in children has also been also advocated for cost problem.^73^ In an American monocentric series of 22 patients from 2002 through 2009, only one child experienced overwhelming post-splenectomy infection after a dog bite.^73^ The authors conclude that earlier surgical consultation for children with chronic ITP may be justified given the high success rate and low morbidity, particularly given the significant complication rate and cost of continued medical treatment.^73^

In conclusion in children splenectomized for ITP pre-vaccinated then risk of late sepsis is present but low, the etiology of capsulated bacteria is rare. Data to make a comparison in children and adults with resistant ITP treated with rituximab are scarce, and inconclusive.^73^–^80^

Liang and Al.^74^ report, in 2012, 11 studies (190 patients) on ITP resistant treated with Rituximab. 78 patients (41.%) experienced adverse events. The most frequently described adverse events were mild allergic reactions and immediate hypersensitivity reaction during rituximab infusion. Four patients developed infections that could be associated with rituximab, including two patients with varicella, one patient with pneumonia, and another patient with life-threatening enterovirus meningoencephalitis. An increased incidence of bacterial infection is also reported in adults treated with rituximab for autoimmune diseases; the presence of diabetes and contemporary use or/and prednisone is a further risk factor.^80^,^81^ Hypogammaglobulinemia has also been reported among adults and children,^76^ although the overall number is unclear and appears to occur with repeated doses and in patients with underlying immune dysfunction. Studies have shown impaired humoral responses to vaccination after rituximab.^82^ However, bacterial infections are reduced in vaccinated patients, and conjugate vaccine should be preferred.^81^,^83^ In conclusion, children and adult with further risk factors should be vaccinated before the treatment with rituximab. This approach is particularly requested if splenectomy is to be considered in the future of the patient.^83^,^84^

In a summary, post-splenectomy infections rate is increased 2–6-fold for first 90 d, and 2.5 (CI, 2.2 to 2.8) more than 365 days after splenectomy in adults with ITP versus indication-matched controls;^7^,^12^ however splenectomy for chronic ITP has a risk of infection not different from subject splenectomized for trauma.^11^ The treatment with rituximab presents a similar risk of bacterial infections. In both conditions, patients should be vaccinated versus *Str. pneumoniae*, *N. meningitidis* and *H. influenza*.^83^–^84^

##### Autoimmune Hemolytic Anemia (AIHA)

Patients with AIHA resistant to steroids can be treated with splenectomy or rituximab.^85^–^87^ Response rates to splenectomy and rituximab seem equivalent even if no prospective study comparing the success rates of both approaches is available.^85^–^87^ So, the side effects are very important in the decision on the choice. In the GIMEMA study, thrombotic events were more frequent in patients who had undergone splenectomy (24% vs 8.7%) and grade 3 pulmonary infections were associated with splenectomy but not with the number of lines of treatment or with the use of rituximab.^86^ In a recent metanalysis^88^ including nineteen studies, among 38 adverse events in 364 patients were reported 4 neutropenias, 18 severe infections, including 1 viral infection, and one *Pneumocystis jiroveci* pneumonia. In conclusion at present in AIHA, the rituximab is increasingly considered the preferred therapy of steroid resistant AIHA.

##### Hemolytic Spherocytosis (HS)

According British guidelines splenectomy should be performed in children with severe HS, considered in those who have moderate disease, and should probably not be performed in those with mild disease.^89^ The two major adverse events are thrombosis and infections.^89^,^90^ Immunization and prophylactic antibiotics could eliminate the increased risk of catastrophic sepsis due to pneumococcus, meningococcus, or haemophilus, and there is evidence that immunization and early use of antibiotics forever have reduced the frequency of positive blood cultures for pneumococcus in children who have had a splenectomy.^89^ Certainly a good compliance of the patients or their relatives to accomplish post-splenectomy infection prophylaxis is fundamental in reducing bacterial infections.^89^,^91^ Accordingly, in a recent report of the American splenectomy in congenital hemolytic anemia registry^91^ among 40 children 2–17 years of age splenectomized for spherocytosis, the infections are relatively low: the rate of early infection was of 2,5 %. Regarding the late adverse events, there were no infections or thrombotic events, and one reoperation (3.1%) over 1 year of follow-up. About 75% of the children were vaccinated, and 97 underwent antibiotics prophylaxis. Furthermore, the most of the patients were submitted to laparoscopic splenectomy.

##### Sickle Cell Anemia. (SCA)

SCA is a hereditary hemolytic anemia due to a homozygous mutation in the gene for β globin, a subunit of adult hemoglobin A (HbA), that results in red blood cell deformity.^92^ It is characterized by recurrent vaso-occlusive episodes, accelerated hemolysis, increased susceptibility to infection, and chronic end-organ damage.^92^–^94^ Acute splenic sequestration crisis (ASSC) is a life-threatening complication of sickle cell disease that occurs secondary to trapping of deformed cells in the splenic vasculature. The result is rapid splenic enlargement, a compensatory elevation of the reticulocyte count, a decrease in hemoglobin level, and potential shock. The mortality for the first episode of ASSC is high particularly in developing countries, approximately 10%, and sequestration can recur in most of the patients.^93^–^96^ These crises can occur as early as the first year of life and can be precipitated by infections.^95^ The consequence of these vaso-occlusive episodes can be the a functional splenectomy, which can occur within the first year of life.^96^ Bacterial infections are one of the main causes of morbidity and mortality in SCD in patients living in both developed or developing countries.^93^–^95^,^97^–^99^ However, the type of bacteria could be different.^97^–^100^ So the utility of vaccination for capsulated bacteria in developing countries has been questioned.^98^ This increased susceptibility is mainly a result of impaired splenic function. However other factors, such as defects in complement activation, micronutrient deficiencies, tissue ischemia and inflammation also contribute.^94^,^96^ Surgical splenectomy seems do not increase the burden of infections while preventing, if complete, further sequestrations and if partial, reducing the recurrence of acute splenic sequestration crises.^100^–^102^ However, there is a lack of evidence that splenectomy improves survival and decreases morbidity in people with SCA.^101^,^102^

Splenectomy has been considered for a long time at high risk of infections, early and late, and of death in children and adult with SCA,^3^,^4^ particularly in children of 4 years or below. Recently, after the introduction of conjugate vaccines^103^,^104^ and prophylactic antibiotics,^105^ splenectomy is considered in developed countries feasible at all age with a moderate risk,^31^,^106^,^107^ which, in any case, is superior to that of other non-malignant hematological diseases.^92^ Data from low-income countries are scarce; splenectomy is considered only in urgency, and then a comparison cannot be done.^108^

##### Thalassemia (Tha)

Splenectomy is recommended in transfusion-dependent Thalassemia to reduce excessive blood consumption and consequent severe iron overload.^109^,^110^ Moreover, a variety of complications such as pulmonary hypertension, silent brain infarcts, venous thrombosis, and sepsis are linked to splenectomy. In particular infections are becoming the leading cause of death in western countries due, in part, to a significant reduction in the number of fatalities from iron-induced cardiac diseases.^109^ Therefore, physicians should keep a guarded approach towards splenectomy because of the its side effects.. At the current time, according the Guidelines for the Management of Transfusion Dependent Thalassaemia,^109^ splenectomy is not recommended standard procedure in transfusion-dependent thalassemia (TDT) subjects. Splenectomy should generally be avoided in Non TDT patients younger than 5 years. Splenectomy should be reserved for cases of:

1. Worsening anemia leading to poor growth and development
2. When transfusion therapy is not possible or iron chelation therapy is unavailable
3. Hypersplenism leading to worsening anemia, leucopenia, or thrombocytopenia and causing clinical problems such as recurrent bacterial infections or bleeding
4. Splenomegaly accompanied by symptoms such as left upper quadrant pain or early satiety
5. Massive splenomegaly (largest dimension >20 cm) with concern about possible splenic rupture

In the past reports a high rate of bacterial infections has been reported in splenectomized patients with thalassemia^4^,^109^,^110^ with the prevalence of sepsis by capsulated bacteria. Nowadays after the widespread adoption of vaccination, the rate of infection is reduced, and most of sepsis is due to Gram- bacteria and *Staphylococcus aureus*.^100^,^111^

Overwhelming post-splenectomy infection (OPSI) by capsulated bacteria have been reported frequently in the past in children (11,6%) with a death of (7,4% ) and also if less commonly in adults, (7.4%) with a death of 3,2%.^4^ A recent Indian study reports a rate of bacterial infection of 17% through 5 years. However, it did not document any OPSI.^111^ It is noteworthy that in this set of patients Malaria was the most frequent post-splenectomy infection Comparisons of the infection rate between thalassemia patients splenectomized or not are rare. A comparative study made in Taiwan^112^ between splenectomized and nonsplenectomized thalassemia patients has been reported in 2003. In this study, the infections were more frequent in splenectomized patients. Notwithstanding the episodic prophylactic vaccination, most of the bacterial infections were Gram negative with a prevalence of *Klebsiella pneumoniae*, which was the most common causative organism in this patient population (10 of 20 isolates). Other pathogens, more frequenly isolated, were *Pseudomonas aeruginosa* and *Vibrio vulnificus.* Recently Chirico et al.^113^ assesses the relationship between infectious events and splenectomized status, HCV infection and serum HMGB1 in 51 adult thalassemia patients. Thirty-six of them (70%) had undergone splenectomy before enrollment. All the patients were vaccinated for capsulated bacteria. During the observational period, 15 patients (29%) reached a primary study endpoint, represented by infectious diseases, requiring hospitalization or parenteral antibiotic administration. Klebsiella infection was documented in 4 cases. Univariate analysis showed that hemoglobin, serum ferritin, splenectomized status and serum HMGB1 values were significantly associated with a primary study endpoint. Results from Cox regression analysis indicated that serum HMGB1, as well as serum ferritin and splenectomized status, predicted a higher risk of infectious disease.

In the last few years the infections by Yersinia, frequently reported in the last decade of the twenty century and associated with an iron overload in transfusion dependent thalassemia,^114^,^115^ are no more signaled. The reduced frequency of capsulated bacterial infections can be attributed to vaccinations and widespread utilization of antibiotic prophylaxis. Furthermore, the use of iron chelator could favor the growth of Klebsiella.^116^ The utility of vaccination and/or preservation of splenic function is undoubtable as demonstrated by an attractive study of Sheikla and Coll.^117^ Two populations of patients from Iraq and Saudi Arabia underwent splenectomy for thalassemia in the same period. All patients from Saudi Arabia were given a preoperative pneumococcal vaccine, polysaccharide pneumococcal vaccine (PPV 23), and underwent total splenectomy after about four weeks. Unfortunately, vaccination was not possible to Iraqi patients, so to this group partial splenectomy was offered to many of these patients as a protective measure against *Streptococcus pneumoniae* infection. Results: A significant difference was found between the total splenectomy fatalities in the two groups. There were five deaths in the 30 enrolled Iraqi patients over four years. One death over a 12-year period was reported in the 22 patients from Saudi Arabia. Partial splenectomy was associated with a dramatic reduction of mortality in the Iraqi patients. None of the 12 patients died during a follow-up period of 4 years. Conclusions: PPV 23 is a powerful prophylactic tool against overwhelming post-splenectomy infection in patients with thalassemia and should be used whenever available. In poor or problematic countries with limited health resources, partial rather than total splenectomy could offer an alternative measure to avoid this fatal complication.

##### No nmalignant Lymphoid Disorders

###### Common variable immunodeficiency disorders

Splenectomy has been used in patients with common variable immunodeficiency disorders (CVID), mainly in the context of refractory autoimmune cytopenia and suspected lymphoma.^118^ Splenectomy proved to be an effective long-term treatment in 75% of CVID patients with autoimmune cytopenia, even in some cases when rituximab had failed. Splenectomy does not worsen mortality in CVID, and adequate immunoglobulin replacement therapy appears to play a protective role in overwhelming post-splenectomy infections. Nine episodes of OPSI including eight cases of bacterial meningitis (two meningococcal, two pneumococcal, one *H. influenzae* and three not stated) and one case of pneumococcal sepsis were reported among 40 patients. IgG trough levels were available for 36 of 40 patients (mean = 8·46 g/l). Six episodes of OPSI occurred prior to Ig replacement therapy, as CVID was not yet diagnosed; one patient made a personal choice not to commence replacement therapy until a later date. Seven of the nine (77·8%) episodes of OPSI occurred within three years of splenectomy, two (22·2%) took place between 4–6 years and none beyond. The annual risk of OPSI was calculated at 2.47% year.

###### Autoimmune lymphoproliferative syndrome

A condition that has characteristics similar to asplenia is found in Autoimmune lymphoproliferative syndrome (ALPS), a rare hereditary disease, caused by impaired FAS-mediated apoptosis of lymphocytes.^119^–^121^ Autoimmune lymphoproliferative syndrome (ALPS) presents in childhood with nonmalignant lymphadenopathy and splenomegaly associated with a characteristic expansion of mature CD4 and CD8 negative or double negative T-cell receptor ab1 T lymphocytes.^119^–^121^ Elevated counts of circulating TCRab1 double-negative CD42CD82 T lymphocyte cells (DN-Ts) are hallmarks of the disease. There is an infiltration of double-negative T-cell (DN-T) in the MZ, which depletes B cells MZ in ALPS patients. These observations suggest that accumulating DN-Ts, trapped within stromal cell meshwork, interfere with correct localization of MZB cells.

An elevated risk of infection was observed in patients with active disease and was associated with a B-cell immunodeficiency characterized by low serum IgM levels, poor production of IgM (but not IgG) anti *Str. pneumoniae* antibodies, low circulating SMB-cells counts, very low circulating MZB, including memory B cells (CD27+/CD19+), MZ B cells (CD27+IgD+/CD19+), and switched memory (SM) B cells (CD27IgD-/CD19+).^120^,^121^

This immunodeficiency strongly correlated with the intensity of lymphoproliferation.^125^ ALPS results in anti-polysaccharide IgM antibody production specific defect with an increased rate of infections from capsulated bacteria. Patients often present with chronic multilineage cytopenias. Cytopenias in these patients can be the result of splenic sequestration as well as autoimmune complications manifesting as autoimmune hemolytic anemia, immune-mediated thrombocytopenia, and autoimmune neutropenia.^119^,^120^ The cytopenias suggested, in the past, to perform frequently splenectomy.^119^ After splenectomy, patients show a significant reduction in anemia (P <0.0001), but neutropenia or thrombocytopenia recur and persist. The rate of invasive bacterial infection in splenectomized patients increases greatly attaining a rate of 30%. A similar risk of severe, post-splenectomy sepsis in ALPS is reported by Price et al.^120^ and by Neven et al.^121^ This risk is much higher than the values of 2%, and 11.6% observed after post trauma splenectomy and in splenectomized thalassemia patients, respectively. Asplenic ALPS patients require vigilance for septicemia because of pneumococcal bacteremia can be fatal. Asplenic ALPS patients can have fatal opportunistic infections and frequently pneumococcal sepsis. All asplenic ALPS patients should preferably remain on long-term antibiotic prophylaxis against pneumococcus using penicillin V or fluoroquinolones, such as levofloxacin. In addition to advising the asplenic patients to wear Medic Alert bracelets, their parents and guardians should be educated about the importance of seeking medical care promptly for a significant febrile illness requiring intravenous antibiotics.^120^,^121^ Recommendations for asplenic ALPS patients include life-long daily antibiotic prophylaxis as well as periodic surveillance and reimmunization against pneumococci using a combination of both 13-valent conjugate (Prevnar-13) and 23-valent polysaccharide.^119^–^121^ The most common bacteria causing septicemia are in the order, *Str. pneumoniae* seen in 70% of patients, *H. influenzae* bacteremia, *N. meningitides* and *Capnocytophaga cynodegmi*. Sepsis can develop notwithstanding antibiotic for prophylaxis and immunization with Prevnar. Clearly overwhelming post-splenectomy sepsis is a major cause of morbidity and mortality.^119^–^121^ Therefore nowadays, avoidance of splenectomy is recommended,^119^–^120^ so, the prognosis for ALPS-FAS is improving and depends, on steroid-sparing management of cytopenias with mycophenolate mofetil or sirolimus, and vigilance for lymphoma.^120^,^121^

## Malignant Hematologic Diseases

Patients splenectomized for malignant hematologic diseases had the highest rates of complication both thrombo-hemorrhagic and infectious.^7^,^8^,^9^,^10^ In the lymphoproliferative diseases, the infectious complications are prevalent;^1^,^4^–^8^,^122^–^124^ on the contrary the thrombo-hemorrhagic complications are prevalent in myeloid neoplasms.^125^–^128^ The condition of malignant hematologic disease per se increases the incidence of bacterial infections.^5^ Regarding *Str. pneumoniae* infection in the United States, the Advisory Committee on Immunization Practices (ACIP) reports the data of the Central Disease Control, (unpublished data, 2012).^129^ An estimated 4,000 deaths occur each year because of *Str. pneumoniae,* primarily among adults*.* The incidence of invasive pneumococcal disease (IPD) ranges from 3.8 per 100,000 among persons aged 18–34 years to 36.4 per 100,000 among those aged ≥65 years. Adults with certain medical conditions also are at increased risk for IPD. For adults aged 18–64 years with hematologic cancer, the rate of IPD in 2010 was 186 per 100,000, and for persons with human immunodeficiency virus (HIV) the rate was 173 per 100,000. The disease rates for adults in these groups can be more than 20 times those for adults without high-risk medical conditions.

### Linfoproliferative Diseases

At present, most of the patients with lymphoproliferative diseases splenectomized are affected by non-Hodgkin Lymphoma (NHL). In the past splenectomy has been utilized for staging Hodgkin Diseases (HD). In splenectomized patients with HD an increase incidence of infection, superior to that found in post-trauma splenectomy has been reported^4^ and vaccination with encapsulated bacteria vaccine is advisable.^122^

Splenectomy in non-Hodgkin lymphoma (NHL), excluding marginal lymphoma, has not a curative intent. It is performed for massive splenomegaly and/or cytopenias to palliate symptoms or in an attempt to improve hematological reserve, so allowing additional medical therapy, or for diagnosis.^2^,^4^–^9^ Although at present most of the patients (50–70 %) splenectomized for hematologic malignant neoplasm in high-income countries are affected by non-Hodgkin lymphoma,^7^–^10^ the data of NHL are not considered separately. There are not investigations separately comparing the rate of infections of NHL patients splenectomized, but the comparison is made between the rate of infections of splenectomized hematological patients with and without a malignant pathology. (Table 1,2)

In these circumstances, the rate of infections is always superior in malignant diseases.^5^–^11^ Thus, given the influence of the basal pathology in determining the complications, to clarify the importance of splenectomy; the comparison should be made with a matched group of NHL patients. It is noteworthy that when the control group is matched-indication the difference in infection risk between splenectomized and not splenectomized is mild (Fig. 3).^5^

Splenectomy remains the treatment of choice in marginal lymphoma of the spleen.^1^,^123^,^124^

One significant concern with splenectomy is the risk of infection from encapsulated organisms and then it is recommended immunization at least two weeks prior to splenectomy. The 4% and 5% patients who underwent splenectomy died from infectious complications in two large series.^123^,^124^ In a recent confrontation between patients treated either with splenectomy or immunochemotherapy the adverse events and, in particular, the infections were more frequent in the follow-up of patients treated with immunochemotherapy.^124^

### Myeloid Neoplasm

Splenectomy rarely is indicated for myeloid neoplasms. Among them, myelofibrosis and monocytic leukemia find more frequently indication for splenectomy.^125^,^126^

#### Myeloproliferative Diseases (MPD)

Among the Myeloproliferative Diseases splenectomy at present is performed for the most in Myelofibrosis because of a huge and/or painful spleen and/or cytopenias.^125^–^128^ Splenectomy is an effective treatment for MPD-related splenic pain and/or cytopenias but is associated with substantial operative morbidity and a mortality ranging from 5 to 18%.^125^–^128^ It is also associated with an increased risk of blast phase transformation,^126^–^128^ and according some studies^127^ to reduced survival.

The recent development of JAK2 inhibitors (e.g. ruxolitinib) as an efficient and safe therapy for patients with MF diminishes the role of splenectomy in everyday management of MF patients.^128^ The main complications are thrombo-hemorrhagic. Infections have been reported as an important complication in the perioperative period ranging from 8,5% to 23% in the different series.^126^–^128^ Rialon et al.^125^ in a series including also patients with MDS report a mortality rate of 18%, whose 13% was due to infections.

## Overwhelming Post-Splenectomy Infection (OPSI)

Although OPSI is reducing after the introduction of vaccinations,^7^,^80^,^130^,^131^ and becoming rare when vaccination are correctly performed,^131^ it remains a possible dangerous event also in the post-anti-pneumococcal vaccination era.^20^, ^37^,^132^ The OPSI can repeat in the same patient. At present it is a minimal proportion of all type of infections in splenectomized patients. In the series of Kyaw et al.^7^ among the 350 (21.2%) patients with severe infection requiring hospitalization only 49 (3.0%) had at least 1 overwhelming infection. Of these, 30 (61.2%) experienced only 1 overwhelming infection, 9 (18.4%) had 2 infections, and 10 (20.4%) had 3 or more severe infections. The incidence of first overwhelming infection was 0.89 per 100 person-years (95% CI, 0.76–1.17). A similar incidence or also lower is reported by others.^131^

OPSI is defined as fulminating sepsis, meningitides or pneumonia triggered mainly by *Str. pneumoniae* followed by *H. influenza*e type B and *N. meningitides*. The risks of OPSI and associated death are highest in the first year after splenectomy, at least among young children, but remain elevated for more than 10 years and probably for life.^37^,^38^,^129^,^130^ OPSI is a medical emergency. Following brief prodromal symptoms such as fever, shivering, myalgia, vomiting, diarrhea, and headache, septic shock develops in just a few hours, with anuria, hypotension, hypoglycemia. A disseminated intravascular coagulation and massive adrenal gland hemorrhage (Waterhouse-Friderichsen syndrome), progressing to multiorgan failure and eventually death can also be present.^37^ The mortality rate is from 50 to 70%, and most death occurs within the first 24 hours; only prompt diagnosis and immediate treatment can reduce mortality.^37^,^132^

Splenectomized children younger than 5 years of age have a greater overall risk of overwhelming infection with an increased death compared with adults.^4^,^5^,^20^,^37^

Physicians must be aware of the potential life-threatening infections in patients who underwent splenectomy and patients should be educated for seeking early care when fever develops.

In patients at risk and with indicative symptoms, prompt initiation of empirical antibiotics is essential.^37^,^132^ Intravenous infusion of third generation cephalosporin (cefotaxime 2 g every 8 h or ceftriaxone 2 g every 12h), combined with gentamicin (5–7 mg/kg every 24 h) or ciprofloxacin (400 mg every 12 h) or vancomycin (1–1.5 g every 12 h). While waiting results of blood culture, bacteria can be visualized by gram staining. An RT-PCR test for simultaneous identification of 3 main encapsulated bacteria (*Str pneumonia, H. influenzae* type B and *N. meningitidis*) is available^20^,^37^,^132^ Taking into account the possibility of gram-negative bacteria in the overwhelming sepsis patient could be started on empirical therapy with carbopenemic antibiotics associated with chinolones and/or vancomycin.

## Prevention of Infections in Patients with an Absent or Dysfunctional Spleen

Education of the patient and its relatives, Vaccination, and antibiotic prophylaxis are the basis to prevent infection by capsulated bacteria and the consequent OPSI.^37^,^129^,^130^–^132^

The patient should be aware of the risk and the necessity of vaccination, which in any case does not preserve from all infections. He should have a clear action for febrile illness, animal bite and planned oversea travel.^131^

### Vaccination

The mainstay of pneumococcal vaccination has, for many years, been the polyvalent polysaccharide pneumococcal vaccine (PPV 23). The PPV23, available since 1983, consist of the capsular polysaccharides of the 23 most prevalent pneumococcal serotypes. It has a coverage of 85–90% of the invasive pneumococcal infections among children and adults.^133^ Its efficacy below five years of age is scarce. Although this polysaccharide vaccine induces an immune response, it does not result in the generation of memory B-cells and long-lived plasma cells. To maintain sufficiently high antibody levels, re-immunization with PPV23 every five years is therefore recommended in hyposplenic and asplenic patients.^133^ Furthermore there is a reduced response to PPV23 in splenectomized patients with hematological diseases.^134^ Conjugate vaccines consist of a polysaccharide covalently linked to a carrier protein (conjugation), this linkage can significantly enhance immunoprotection against the polysaccharide by inducing a T-cell-dependent immune response. Conjugate vaccines are highly immunogenic in infants as young as two months of age, provide higher antibody titers and induce immunological memory.^129^,^135^

The first heptavalent pneumococcal polysaccharide-protein conjugate vaccine (PCV7) was introduced in USA 2000 and Europe in 2006.^135^–^138^ This conjugate vaccine leads to T-cell dependent induction of antibodies and immunological memory. The seven serotypes, included in the conjugate vaccine, handle 64% of the invasive pneumococcal infections in young children (<2 years) of the Netherlands.^135^ The inclusion of conjugated pneumococcal polysaccharide vaccines might be of additional value in the vaccination schedule for asplenic patients because of their high immunogenicity.^136^–^139^ A strong serological response was found in splenectomized patients within the first five years after pneumococcal vaccination by PCV7. Nevertheless, post-vaccine pneumococcal sepsis was still diagnosed in 3.3% of splenectomized survivors. However, sepsis and death were found for the most in patients with hematologic malignancies, frequently with severe neutropenia.^140^

Since 2010, two improved pneumococcal conjugate vaccines (PCVs) received market authorization in many countries, including in the US and the EU.^138^ These vaccines cover the seven serotypes included in the PCV7 vaccine, and additional serotypes responsible for an increasing proportion of IPD. Specifically, PCV10 (“SynflorixTM”, GSK) contains additional antigens from serotypes 1, 5 and 7F.^133^–^135^,^138^ The manufacturer claims a high protective effect against diseases not only due to pneumococcal serotypes but also against disease due to non-typeable *H. influenzae* PCV13 (“Prevnar13TM”, Pfizer) contains antigens from serotypes 1, 3, 5, 6A, 7F and 19A in addition to the PCV7serotypes.^138^ The 13-valent pneumococcal conjugate vaccine (PCV13) has replaced in the last few year the PCV7. As predicted, PCV13 is more immunogenic than PPV23 albeit with a more limited repertoire and is highly effective in preventing invasive disease caused by the 13 serotypes included in the vaccine.^138^,^139^

At present, the PCV 13 has been added to PPV 23 in all guidelines of high-income countries in children age.^129^,^140^–^142^ According the UK guidelines of 2011, at present, for older children and adults who may or may not have received previous PCV there is insufficient evidence to recommend a change in policy from PPV to PCV either for primary immunization or for boosting. Similarly, in the United States, guidelines from the Centers for Diseases Control and Prevention (CDC), published in 1997 and updated in 2010, recommended the use of the 23-valent pneumococcal polysaccharide vaccine (PPV23) in adults with anatomical or functional asplenia and revaccination after 5 years.^140^ Whether patients should be recommended pneumococcal polysaccharide vaccine(PPV) or pneumococcal conjugate vaccine (PCV) and the possible benefits of repeated vaccinations be the subject of a current debate in Europe.^137^–^138^ However, in USA, the high rate of invasive pneumococcal diseases (IPD) found through 2010 among adults aged 18–64 years with hematologic cancer induced the Advisory Committee on Immunization Practices (ACIP) on 20 June 2012 to extend routine use of 13-valent pneumococcal conjugate vaccine (PCV13; Prevnar 13, Wyeth Pharmaceuticals, Inc., a subsidiary of Pfizer, Inc.) to adults aged ≥19 years with functional or anatomic asplenia, other than to other immunocompromised conditions, cerebrospinal fluid (CSF) leaks, or cochlear implants.^129^ This decision was made by considering that 50% of IPD cases among immunocompromised adults in 2010 were caused by serotypes contained in PCV13; an additional 21% were caused by serotypes only contained in PPSV23 (CDC, unpublished data, 2011). Consequently the PCV13 should be administered to eligible adults in addition to the 23-valent pneumococcal polysaccharide vaccine (PPSV23; Pneumovax 23, Merck & Co. Inc.), the only vaccine currently recommended for these groups of adults in most European guidelines. The recent paper of Nived^139^ demonstrate after PVC 13 vaccination high levels of pneumococcal serotype-specific antibodies in the previous PPV23 vaccinated group, demonstrating that PCV 13 can be used as a booster dose in asplenic patients with previous PPV23 vaccination. High levels of serotype-specific IgG concentration ≥0.35 mg/mL were observed in previous PPV23 vaccinated but PCV-naïve asplenic patients for serotypes 1, 3, 4, 5, 7F, 18C,19A, 19F, and 23F.^137^ Safety and immunogenicity of sequential administration have been demonstrated in older people^144^,^145^ and recently its safety and efficacy has been confirmed by a large trial including nonhematological patients.^146^

Polysaccharide vaccines and conjugate vaccines are both available against *Haemophilus influenzae B* and *Neisseria meningitidis*.^147^–^150^ Conjugate vaccines activate a superior immune response compared with polysaccharide vaccines and shows efficacy in children 2–4 years old as well in older adults. Thus, conjugate vaccines should be used preferentially whenever possible not in substitution but also of polysaccharide vaccine.

A quadrivalent meningococcal diphtheria toxoid conjugate vaccine (Menactra®, Sanofi Pasteur) (MCV4) including serogroups A, C, Y, and W was licensed for use in 2005 by the US FDA and in 2007 licensure was approved in Canada, and in the Arab Gulf countries.^147^ This vaccine should be utilized in Arab countries, where the serotype W is particularly frequent.^147^ It does not cover against the strain B, which is the predominant cause of invasive meningococcal disease in most of Europe and Australia countries, especially where serogroup C vaccination is part of routine recommendations.^148^ However, at present is available also a vaccine against the strain B. The multicomponent meningococcal B vaccine, 4CMenB (Bexsero, Novartis Vaccines and Diagnostics), recently approved in Europe and Australia, contains three surface-exposed recombinant proteins (fHbp, NadA, and NHBA) and New Zealand strain outer membrane vesicles (NZ OMV) with PorA 1.4 antigenicity.^148^

In our opinion the modality of vaccination should follow the scheme adopted by the Canadian Paediatric Society,^143^ integrated by the recent recommendation of ACIP (Table 4). 129

In programmed splenectomy, the vaccine should be administered two weeks before intervention and in urgency two weeks afterward both in adults and children.

Both the conjugated 13-valent conjugate pneumococcal vaccine and the 23-valent polysaccharide vaccine should be utilized in the prevention of *Streptococcus pneumoniae* infection. In pneumococcal vaccine-naïve persons: Adults aged ≥19 years with immunocompromised conditions, functional or anatomic asplenia, CSF leaks, or cochlear implants, and who have not previously received PCV13 or PPSV23, should be given a dose of PCV13 first, followed by a dose of PPSV23 at least 8 weeks later. Subsequent doses of PPSV23 should follow current PPSV23 recommendations for adults at high risk. Specifically, a second PPSV23 dose is recommended five years after the first PPSV23 dose for persons aged 19–64 years with functional or anatomic asplenia and for persons with immunocompromised conditions. Additionally, those who received PPSV23 before age 65 years for any indication should be given another dose of the vaccine at age 65 years, or later if at least five years have elapsed since their previous PPSV23 dose. In the previous vaccinated with PPSV23: Adults aged ≥19 years with immunocompromised conditions, functional or anatomic asplenia, who previously have received ≥1 doses of PPSV23 should be given a PCV13 dose ≥1 year after the last PPSV23 dose was received. For those who require additional doses of PPSV23, the first such dose should be given no sooner than eight weeks after PCV13 and at least five years after the most recent dose of PPSV23.

- In prevention of *Haemophilus influenzae*: type b conjugate vaccine; specific recommendations vary by age.
- In prevention of *Neisseria meningitidis*: conjugate quadrivalent meningococcal vaccine (MCV4) should be utilized. Experience with the multi-component meningococcal B vaccine is scarce, however epidemiological studies suggest its utilization in Europe and Australia.

Apart from the scarce compliance,^149^ some patients remain unvaccinated, despite this double vaccination and a true vaccine failure also contribute to pneumococcal infection.

Failure to mount an antibody response may be genetically determined but is also more frequent in older patients and those splenectomized for hematological malignancies.^150^,^151^ The failure to respond to immunization can be demonstrated by the absent rise in titer of the anti-pneumococcal antibody.^133^,^134^,^136^ A surge of non-vaccine serotypes could be another cause of failure of vaccination as described after the addition of pneumococcal protein conjugate vaccine (PCV7).^152^

### Antibiotic prophylaxis

Lifelong antibiotics are recommended for immunosuppressed patients, and for at least two years after splenectomy for all other patients. Further, patients are advised to keep an emergency supply of antibiotics for the event of febrile illness. Oral penicillins remain the prophylactic drugs of choice in areas with low pneumococcal resistance. Specialist microbiological advice should be sought where this is not the case or for travel abroad. In patients with confirmed penicillin allergy, an appropriate macrolide may be substituted depending on local epidemiology.

## Concluding Remarks

Splenectomy, even if the incidence of OPSI is reducing in high-income countries for the widespread pneumococcal vaccination, also represent today’s an important risk factor for infections. The introduction of conjugate vaccines also in the older population could induce a further reduction of sepsis from encapsulated bacteria. The case reported at the incipit of this review presented meningitis with a culture of liquor positive for *Str. pneumoniae*. The addition of a conjugate vaccine could have increase the immunological response reducing the risk of infection. In the high-income countries, the antipneumococcal vaccination is adequate, at least in terms of primary vaccination with Pneumovax, but conjugate vaccines have not been introduced so far in most of the countries. In contrast, vaccination against *N. meningitidis* serogroups A + C was insufficient and introduction of vaccination against B serotype is warranted. There is a need to improve the awareness among healthcare professionals of the greatly increased risk of severe infection with encapsulated bacteria post-splenectomy and how these infections, in particular, overwhelming post-splenectomy infection, can be prevented. However, at present gram- negative sepsis are prevalent. Further work is required to characterize these infections and determine whether or not they were related to asplenia.

OPSI continue to be described in 1–1.5 patients/year also in vaccinated patients, but *Streptococcus pneumoniae*, which was in the past the major cause of morbidity and mortality among such patients, has become infrequent as a cause of infections, at least in European series. Poorly controlled iron overload can be the cause of Gram-negative infections that are still frequently diagnosed in post-splenectomy patients for congenital hemoglobin disorders. This information needs to be taken into account when a splenectomized patient presents with fever and/or sepsis. At the first indication of systemic infection (high fever) all patients should have access to primary care and start urgent treatment with appropriate antibiotics, (in general treatment with a third generation cephalosporin, either alone or in combination with other antibiotics that are active against Gram-negative pathogens, should be promptly initiated in order to save the patient’s life). In patients taking prophylaxis treatment should be from an antibiotic class likely to be non-cross resistant. Choice of antibiotic should be made concerning appropriate microbiological advice and local protocols. The importance of the primitive disease remains fundamental in determining the rate and the severity of infections and the overall survival.

## Figures and Tables

**Table 1:** Causes of splenectomies in different series.

Authors, year (references)	Total Number	Trauma N°( %)	Hematologic Diseases N°(%)		Others N°(%)	
			Malignant	Nonmalignant	Malignant	Nonmalignant
Bisharat, 2001 (4)	6942	3122 (45.0)	731(10.5)	2416 (34.8)	673 (21)	
Kyaw, 2006 (7)	1648	**271**	143	447	530	246
Edgren,2014 (8)	7158	990 (13.8)	691(9.7)	1485 (20.7)	2476 (34.6)	1516(21.2)
Dendle, 2012 (6)	2472	635	269	583	497	488
Khamechian 2013, (28)	99	75 (75.8)		9 (9.1)	6(6)	9(9.1)

**Table 2:** Percentage of splenectomy in the different hematologic pathologies, according different countries and times.

AUTHORS (Ref.) Country, year N° cases	NONMALIGNANT DISEASES N° (%)						MALIGNANT DISEASES N° (%)		
	ITP	AIHA	SPHERO	THAL	SCA	Others	Lymphoma	MPD/MDS	OTHER
Bisharat (4) Israel, 2001 3147	484 (15,4)		1432 (45,5)	293 (9.3)	207 (6.5)		731 (23.2)		
Bagrodia (10) USA, 2014 1715	988 (57.6)	153 (8.9)	53 (3.1)			23 (2)	312 (18.2)	12 (1.0)	59 (3.4)
Machado (30) Oman (2009) 150	12 (8)	2(1,3)	6 (4)		96 (64))				
Casaccia (24) Italy (2010) 676	246 (36.4)	12 (1.7)	62 (9.2)	30 (4.4)		134 (18.8)	178 (26.3)	12(1.7)	26 (3.8)
Bickembach (9) USA, 2013 381	17 (4.5)	10 (2.6)	3 (0.8)			34 (8.9)	197 (51.6)	37 (9.7)	83 (21.8)
Thomsen (5) Denmark (2009) 1156	269 (23,2)		145(12.5)			454 (39.2)	288 (24,9)		
Edgren (8) Sweeden, 2014 2176	1485 (68.2)						691(31.8)		

**Table 3:** Rate of severe infections in patients splenectomized for different diseases.

Authors (ref.)		Nonmalignant Diseases					Malignant Diseases	
	Trauma	ITP	AIHA	Sphero	Thal.	SCA	Lymph.	MPD/MDS
Bisharat (4)	2.3%	2.1%		3.1%	8.2%	7.3%	4.1%	
Thomsen (5)	2.5 (RR)	4.0		4.0			5.8	
Rice (91)				2.5%		10%		
Dendle (6)	3.12%	4.81%					13.26	
Edgren (8)	3.8 (SIR)	8.1(SIR)					20 (SIR)	

RR= Relative Risk; SIR= Standardized Incidence Ratio ; Sphero= Spherocytosis; Lymph= Lymphoma; MDP= Myloproliferative Diseases; MDS= Myelodysplastic Syndromes; ITP= Immune thrombocytopenic Purpura; AIHA= Autoimmune Hemolytic Anemia; Thal.= Thalassemia; SCA= Sickle Cell Anemia.

**Table 4:** Dose and time administration of Vaccines.

Bacteria	*Str. pneumoniae*	*N. meningitidis*	*H. influenzae* type b (HIB)
Vaccines	Conjugated 13-valent pneumococcal vaccine (PCV13) 23-valent polysaccharide vaccine (PPV23)	conjugate quadrivalent meningococcal vaccine (MCV4). Vaccine serotype B (4CMenB)	Anti HIB conjugate vaccine
Administration Time	PCV13.Children 12–24 ms : 4 doses at 2, 4, 6 and 12 to 15 m. . Pts >24 ms: one dose . PPV23.Pts>24 ms: one dose+booster dose after 5 ys	MCV4. Children >12 ms: 4 doses at 2, 4, 6, 12–15 ms. Pts>12 ms: 2 doses 8 wks apart All Pts revaccinated every 5 ys 4CMenB. should be given to all asplenic patients including infants.	AntiHIB. Children <18 ms: 3 doses at 2, 4, 6 ms, booster dose, 18 ms Pts>5yrs: one dose to be repeated in case of infection.

Abbreviations: Pts= patients; ms= months; ys= years

## References

[1] Olszewski AJ, Ali S (2014). Comparative outcomes of rituximab-based systemic therapy and splenectomy in splenic marginal zone lymphoma. Ann Hematol.

[2] Katz SC, Pachter HL (2006). Indications for splenectomy. Am Surg.

[3] Weledji EP (2014). Benefits and risks of splenectomy. Int J Surg.

[4] Bisharat N, Omari H, Lavi I, Raz R (2001). Risk of infection and death among post-splenectomy patients. J Infect.

[5] Thomsen RW, Schoonen WM, Farkas DK, Riis A, Jacobsen J, Fryzek JP, Sørensen HT (2009). Risk for hospital contact with infection in patients with splenectomy: a population-based cohort study. Ann Intern Med.

[6] Dendle C, Sundararajan V, Spelman T, Jolley D, Woolley I (2012). Splenectomy sequelae: an analysis of infectious outcomes among adults in Victoria. Med J Aust.

[7] Kyaw MH, Holmes EM, Toolis F, Wayne B, Chalmers J, Jones IG, Campbell H (2006). Evaluation of severe infection and survival after splenectomy. Am J Med.

[8] Edgren G, Almqvist R, Hartman M, Utter G (2014). Splenectomy and the Risk of Sepsis: A Population-Based Cohort Study. Ann Surg.

[9] Bickenbach KA, Gonen M, Labow DM, Strong V, Heaney ML, Zelenetz AD, Coit DG (2013). Indications and efficacy of splenectomy for haematological disorders. Br J Surg.

[10] Magrodia N, Button AM, Spanheimer PM, Belding-Schmitt ME, Rosenstein LJ, Mezhir JJ (2014). Morbidity and mortality following elective splenectomy for benign and malignant hematologic conditions: analysis of the American College of Surgeons National Surgical Quality Improvement Program data. JAMA Surg.

[11] Kristinsson SY, Gridley G, Hoover RN, Check D, Landgren O (2014). Long-term risks after splenectomy among 8,149 cancer-free American veterans: a cohort study with up to 27 years follow-up. Haematologica.

[12] Rodeghiero F, Ruggeri M (2012). Short- and long-term risks of splenectomy for benign haematological disorders: should we revisit the indications?. Br J Haematol.

[13] Rubin LG, Schaffner W (2014). Clinical practice. Care of the asplenic patient. N Engl J Med.

[14] Aubrey-Bassler FK, Sowers N (2012). 613 cases of splenic rupture without risk factors or previously diagnosed disease: a systematic review. BMC Emerg Med.

[15] Rabie ME, Hashemey AA, El Hakeem I, Al Hakamy MA, Obaid M, Al Skaini M, Shabbir G, Al Sareii S, Hussain MN (2010). Spontaneous rupture of malarial spleen: report of two cases. Mediterr J Hematol Infect Dis.

[16] Al-Salem AH (2013). Massive splenic infarction in children with sickle cell anemia and the role of splenectomy. Pediatr Surg Int.

[17] kKubber MM, Kroft LJ, de Groot B (2013). Non-traumatic splenic rupture in a patient on oral anticoagulation. Int J Emerg Med.

[18] Maria V, Saad AM, Fardellas I (2013). Spontaneous spleen rupture in a teenager: an uncommon cause of acute abdomen. Case Rep Med.

[19] Cesaro S, Marson P, Gazzola MV, De Silvestro G, Destro R, Pillon M, Calore E, Messina C, Zanesco L (2002). The use of cytokine-stimulated healthy donors in allogeneic stem cell transplantation. Haematologica.

[20] Di Sabatino A, Carsetti R, Corazza GR (2011). Post-splenectomy and hyposplenic states. Lancet.

[21] Harbrecht BG, Franklin GA, Miller FB, Richardson JD (2008). Is splenectomy after trauma an endangered species?. Am Surg.

[22] Fung HC, Nademanee AP (2002). Approach to Hodgkin’s lymphoma in the new millennium. Hematol Oncol.

[23] Mahévas M, Fain O, Ebbo M, Roudot-Thoraval F, Limal N, Khellaf M, Schleinitz N, Bierling P, Languille L, Godeau B, Michel M (2014). The temporary use of thrombopoietin-receptor agonists may induce a prolonged remission in adult chronic immune thrombocytopenia. Results of a French observational study. Br J Haematol.

[24] Casaccia M, Torelli P, Pasa A, Sormani MP, Rossi E, IRLSS Centers (2010). Putative predictive parameters for the outcome of laparoscopic splenectomy: a multicenter analysis performed on the Italian Registry of Laparoscopic Surgery of the Spleen. Ann Surg.

[25] Sheikha AK, Salih ZT, Kasnazan KH, Khoshnaw MK, Al-Maliki T, Al-Azraqi TA, Zafer MH (2007). Prevention of overwhelming postsplenectomy infection in thalassemia patients by partial rather than total splenectomy. Can J Surg.

[26] National Hospital Discharge Survey.

[27] Sickle Cell Association of America Research and screening.

[28] Khamechian T, Alizargar J, Farzanegan M (2013). Pattern of splenectomy indications inkashan shahid-beheshti hospital: a 5-year study. Arch Trauma Res.

[29] Deodhar M, Kakkar N (2004). An audit of splenectomies in a teaching hospital in North India. Are postsplenectomy guidelines being complied with?. J Clin Pathol.

[30] Machado NO, Grant CS, Alkindi S, Daar S, Al-Kindy N, Al Lamki Z, Ganguly SS (2009). Splenectomy for haematological disorders: a single center study in 150 patients from Oman. Int J Surg.

[31] Alufohai E, Odusanya OO (2006). Splenectomy in a rural surgical practice. Niger J Clin Pract.

[32] Wang X, Li Y, Crook N, Peng B, Niu T (2013). Laparoscopic splenectomy: a surgeon’s experience of 302 patients with analysis of postoperative complications. Surg Endosc.

[33] Hayashi H, Beppu T, Shirabe K, Maehara Y, Baba H (2014). Management of thrombocytopenia due to liver cirrhosis: a review. World J Gastroenterol.

[34] Barmparas G, Lamb AW, Lee D, Nguyen B, Eng J, Bloom MB, Ley EJ (2015). Postoperative infection risk after splenectomy: A prospective cohort study. Int J Surg.

[35] Boyle S, White RH, Brunson A, Wun T (2013). Splenectomy and the incidence of venous thromboembolism and sepsis in patients with immune thrombocytopenia. Blood.

[36] Holdsworth RJ, Irving AD, Cuschieri A (1991). Postsplenectomy sepsis and its mortality rate: actual versus perceived risks. Br J Surg.

[37] Sinwar PD (2014). Overwhelming post splenectomy infection syndrome - review study. Int J Surg.

[38] Ejstrud P, Kristensen B, Hansen JB, Madsen KM, Schønheyder HC, Sørensen HT (2000). Risk and patterns of bacteraemia after splenectomy: a population-based study. Scand J Infect Dis.

[39] Yu RK, Shepherd LE, Rapson DA (2000). Capnocytophaga canimorsus, a potential emerging microorganism in splenectomized patients. Br J Haematol.

[40] Sica S, Di Mario A, Salutari P, d’Onofrio G, Antinori A, Chiusolo P, Leone G (1995). Morganella morganii pericarditis after resolvent splenectomy for immune pancytopenia following allogeneic bone marrow transplantation for acute lymphoblastic leukemia. Clin Infect Dis.

[41] Demar M, Legrand E, Hommel D, Esterre P, Carme B (2004). Plasmodium falciparum malaria in splenectomized patients: two case reports in French Guiana and a literature review. Am J Trop Med Hyg.

[42] Rosner F, Zarrabi MH, Benach JL (1984). Babesiosis in splenectomized adults. Review of 22 reported cases. Am J Med.

[43] Stowell CP, Gelfand JA, Shepard JA (2007). Case records of the Massachusetts General Hospital. Case 17–2007. A 25-year-old woman with relapsing fevers and recent onset of dyspnea. N Engl J Med.

[44] Mebius RE, Kraal G (2005). Structure and function of the spleen. Nat Rev Immunol.

[45] Zandvoort A, Timens W (2002). The dual function of the splenic marginal zone:essential for initiation of anti-TI-2 responses but also vital in the general first-line defense against blood-borne antigens. Clin Exp Immunol.

[46] Kruetzmann S, Rosado MM, Weber H (2003). Human immunoglobulin M memory B cells controlling Streptococcus pneumoniae infections are generated in the spleen. J Exp Med.

[47] Weller S, Braun MC, Tan BK (2004). Human blood IgM “memory” B cells are circulating splenic marginal zone B cells harboring a prediversified immunoglobulin repertoire. Blood.

[48] Carsetti R, Pantosti A, Quinti I (2006). Impairment of the antipolysaccharide response in splenectomized patients is due to the lack of immunoglobulin M memory B cells. J Infect Dis.

[49] Moens L, Wuyts M, Meyts I, De Boeck K, Bossuyt X (2008). Human memory B lymphocyte subsets fulfill distinct roles in the anti-polysaccharide and antiprotein immune response. J Immunol.

[50] Wasserstrom H, Bussel J, Lim LC, Cunningham-Rundles C (2008). Memory B cells and pneumococcal antibody after splenectomy. J Immunol.

[51] Rosado MM, Gesualdo F, Marcellini V, Di Sabatino A, Corazza GR, Smacchia MP, Nobili B, Baronci C, Russo L, Rossi F, Vito RD, Nicolosi L, Inserra A, Locatelli F, Tozzi AE, Carsetti R Preserved antibody levels and loss of memory B cells against pneumococcus and tetanus after splenectomy: tailoring better vaccination strategies. Eur J Immunol.

[52] Koppel EA, Saeland E, de Cooker DJ, van Kooyk Y, Geijtenbeek TB (2005). DC-SIGN specifically recognizes Streptococcus pneumonia serotypes 3 and 14. Immunobiology.

[53] Bronte V, Pittet MJ (2013). The spleen in local and systemic regulation of immunity. Immunity.

[54] den Haan JM, Kraal G (2012). Innate immune functions of macrophage subpopulations in the spleen. J Innate Immun.

[55] Ram S, Lewis LA, Rice PA (2010). Infections of people with complement deficiencies and patients who have undergone splenectomy. Clin Microbiol Rev.

[56] Kang YS, Do Y, Lee HK, Park SH, Cheong C, Lynch RM, Loeffler JM, Steinman RM, Park CG (2006). A dominant complement fixation pathway for pneumococcal polysaccharides initiated by SIGN-R1 interacting with C1q. Cell.

[57] Prabagar MG, Do Y, Ryu S, Park JY, Choi HJ, Choi WS, Yun TJ, Moon J, Choi IS, Ko K, Ko K, Young Shin C, Cheong C, Kang YS (2013). SIGN-R1, a C-type lectin, enhances apoptotic cell clearance through the complement deposition pathway by interacting with C1q in the spleen. Cell Death Differ.

[58] Heuer M, Taeger G, Kaiser GM, Nast-Kolb D, Kühne CA, Ruchholtz S, Lefering R, Paul A, Lendemans S, Trauma Registry of DGU (2010). No further incidence of sepsis after splenectomy for severe trauma: a multi-institutional experience of the trauma registry of the DGU with 1,630 patients. Eur J Med Res.

[59] Wiseman J, Brown CV, Weng J, Salim A, Rhee P, Demetriades D (2006). Splenectomy for trauma increases the rate of early postoperative infections. Am Surg.

[60] Boni L, Benevento A, Rovera F, Dionigi G, Di Giuseppe M, Bertoglio C, Dionigi R (2006). Infective complications in laparoscopic surgery. Surg Infect (Larchmt).

[61] Neunert CE (2013). Current management of immune thrombocytopenia. Hematology Am Soc Hematol Educ Program.

[62] Leone G, Larocca LM, Scribano D, Storti S, Caputo G, Landolfi R (1986). T lymphocyte subsets and platelet-associated IgG in idiopathic thrombocytopenic purpura: effect of splenectomy. Acta Haematol.

[63] Vianelli N, Palandri F, Polverelli N, Stasi R, Joelsson J, Johansson E, Ruggeri M, Zaja F, Cantoni S, Catucci AE, Candoni A, Morra E, Björkholm M, Baccarani M, Rodeghiero F (2013). Splenectomy as a curative treatment for immune thrombocytopenia: a retrospective analysis of 233 patients with a minimum follow up of 10 years. Haematologica.

[64] Cooper N, Evangelista ML, Amadori S, Stasi R (2007). Should rituximab be used before or after splenectomy in patients with immune thrombocytopenic purpura?. Curr Opin Hematol.

[65] Khellaf M, Charles-Nelson A, Fain O, Terriou L, Viallard JF, Cheze S, Graveleau J, Slama B, Audia S, Ebbo M, Le Guenno G, Cliquennois M, Salles G, Bonmati C, Teillet F, Galicier L, Hot A, Lambotte O, Lefrère F, Sacko S, Kengue DK, Bierling P, Roudot-Thoraval F, Michel M, Godeau B (2014). Safety and efficacy of rituximab in adult immune thrombocytopenia: results from a prospective registry including 248 patients. Blood.

[66] Fianchi L, Rossi E, Murri R, De Stefano V, Pagano L, Leone G (2007). Severe infectious complications in a patient treated with rituximab for idiopathic thrombocytopenic purpura. Ann Hematol.

[67] Moulis G, Lapeyre-Mestre M, Mahévas M, Montastruc JL, Sailler L (2015). Need for an improved vaccination rate in primary immune thrombocytopenia patients exposed to rituximab or splenectomy. A nationwide population-based study in France. Am J Hematol.

[68] Moulis G, Sailler L, Sommet A, Lapeyre-Mestre M, Derumeaux H, Adoue D (2014). Rituximab versus splenectomy in persistent or chronic adult primary immune thrombocytopenia: an adjusted comparison of mortality and morbidity. Am J Hematol.

[69] Gonzalez-Porras JR, Escalante F, Pardal E, Sierra M, Garcia-Frade LJ, Redondo S, Arefi M, Aguilar C, Ortega F, de Cabo E, Fisac RM, Sanz O, Esteban C, Alberca I, Sanchez-Barba M, Santos MT, Fernandez A, Gonzalez-Lopez TJ, Grupo de Trombosis y Hemostasia de Castilla y León (2013). Safety and efficacy of splenectomy in over 65-yrs-old patients with immune thrombocytopenia. Eur J Haematol.

[70] Aronis S, Platokouki H, Avgeri M (2004). Retrospective evaluation of long-term efficacy and safety of splenectomy in chronic idiopathic thrombocytopenic purpura in children. Acta Paediatr.

[71] Kuhne T, Blanchette V, Buchanan GR (2007). Splenectomy in children with idiopathic thrombocytopenic purpura: a prospective study of 134 children from the intercontinental childhood ITP Study Group. Pediatr Blood Cancer.

[72] Aladjidi N, Santiago R, Pondarré C (2012). Revisiting splenectomy in childhood chronic immune thrombo- cytopenia at the era of new therapies:the French experience. J Blood Disorders Transf.

[73] Gwilliam NR, Lazar DA, Brandt ML, Mahoney DH, Wesson DE, Mazziotti MV, Nuchtern JG, Lee TC (2012). An analysis of outcomes and treatment costs for children undergoing splenectomy for chronic immune thrombocytopenia purpura. J Pediatr Surg.

[74] Liang Y, Zhang L, Gao J, Hu D, Ai Y (2012). Rituximab for children with immune thrombocytopenia: a systematic review. PLoS One.

[75] Zhou H, Xu M, Qin P, Zhang HY, Yuan CL, Zhao HG, Cui ZG, Meng YS, Wang L, Zhou F, Wang X, Li DQ, Bi KH, Zhu CS, Guo CS, Chu XX, Wu QC, Liu XG, Dong XY, Li J, Peng J, Hou M (2015). A multicenter randomized open-label study of rituximab plus rhTPO vs rituximab in corticosteroid-resistant or relapsed ITP. Blood.

[76] Ansari Sh, Rostami T, Yousefian S, Kiumarsi A, Miri-Aliabad G, Ramim T (2014). Rituximab efficacy in the treatment of children with chronic immune thrombocytopenic purpura. Pediatr Hematol Oncol.

[77] Cooper N (2014). A review of the management of childhood immune thrombocytopenia: how can we provide an evidence-based approach?. Br J Haematol.

[78] Matsubara K, Takahashi Y, Hayakawa A, Tanaka F, Nakadate H, Sakai M, Maeda N, Oka T, Ishii E, Bessho F, Morimoto T, Goto H, Hashii Y, Hatakeyama N, Shirahata A, Imaizumi M (2014). Long-term follow-up of children with refractory immune thrombocytopenia treated with rituximab. Int J Hematol.

[79] Moulis G, Sailler L, Sommet A, Lapeyre-Mestre M, Derumeaux H, Adoue D (2014). Rituximab versus splenectomy in persistent or chronic adult primary immune thrombocytopenia: an adjusted comparison of mortality and morbidity. Am J Hematol.

[80] Patel VL, Mahévas M, Lee SY, Stasi R, Cunningham-Rundles S, Godeau B, Kanter J, Neufeld E, Taube T, Ramenghi U, Shenoy S, Ward MJ, Mihatov N, Patel VL, Bierling P, Lesser M, Cooper N, Bussel JB (2012). Outcomes 5 years after response to rituximab therapy in children and adults with immune thrombocytopenia. Blood.

[81] Heusele M, Clerson P, Guery B, Lambert M, Launay D, Lefevre G, Morell-Dubois S, Maillard H, Le Gouellec N, Hatron PY, Hachulla E (2014). Risk factors for severe bacterial infections in patients with systemic autoimmune diseases receiving rituximab. Clin Rheumatol.

[82] Crnkic Kapetanovic M, Saxne T, Jönsson G, Truedsson L, Geborek P (2013). Rituximab and abatacept but not tocilizumab impair antibody response to pneumococcal conjugate vaccine in patients with rheumatoid arthritis. Arthritis Res Ther.

[83] Svensson M, Dahlin U, Kimby E (2012). Better response with conjugate vaccine than with polysaccaride vaccine 12 months after rituximab treatment in lymphoma patients. Br J Haematol.

[84] Moulis G, Lapeyre-Mestre M, Mahévas M, Montastruc JL, Sailler L (2014). Need for an improved vaccination rate in primary immune thrombocytopenia patients exposed to rituximab or splenectomy. A nationwide population-based study in France. Am J Hematol.

[85] Zanella A, Barcellini W (2014). Treatment of autoimmune hemolytic anemias. Haematologica.

[86] Barcellini W, Fattizzo B, Zaninoni A (2014). Clinical heterogeneity and predictors of outcome in primary autoimmune hemolytic anemia: a GIMEMA study of 308 patients. Blood.

[87] Dierickx D, Kentos A, Delannoy A (2015). The role of rituximab in adults with warm antibody autoimmune hemolytic anemia. Blood.

[88] Reynaud Q, Durieu I, Dutertre M, Ledochowski S, Durupt S, Michallet AS, Vital-Durand D, Lega JC (2015). Efficacy and safety of rituximab in auto-immune hemolytic anemia: A meta-analysis of 21 studies. Autoimmun Rev.

[89] Bolton-Maggs PHB, Stevens RF, Dodd NJ, Lamont G, Tittensor P, King MJ (2004). Guidelines for the diagnosis and management of hereditary spherocytosis. British Journal of Haematology.

[90] Schilling RF (2009). Risks and benefits of splenectomy versus no splenectomy for hereditary spherocytosis--a personal view. Br J Haematol.

[91] Rice HE, Englum BR, Rothman J, Leonard S, Reiter A, Thornburg C, Brindle M, Wright N, Heeney MM, Smithers C, Brown RL, Kalfa T, Langer JC, Cada M, Oldham KT, Scott JP, St Peter S, Sharma M, Davidoff AM, Nottage K, Bernabe K, Wilson DB, Dutta S, Glader B, Crary SE, Dassinger MS, Dunbar L, Islam S, Kumar M, Rescorla F, Bruch S, Campbell A, Austin M, Sidonio R, Blakely ML, Splenectomy in Congenital Hemolytic Anemia (SICHA) Consortium (2015). Clinical outcomes of splenectomy in children: report of the splenectomy in congenital hemolytic anemia registry. Am J Hematol.

[92] De Franceschi L (2009). Pathophisiology of sickle cell disease and new drugs for the treatment. Mediterr J Hematol Infect Dis.

[93] Booth C, Inusa B, Obaro SK (2010). Infection in sickle cell disease: a review. Int J Infect Dis.

[94] Sobota A, Sabharwal V, Fonebi G, Steinberg M (2015). How we prevent and manage infection in sickle cell disease. Br J Haematol.

[95] Ahmed SG (2011). The role of infection in the pathogenesis of vaso-occlusive crisis in patients with sickle cell disease. Mediterr J Hematol Infect Dis.

[96] Brousse V, Buffet P, Rees D (2014). The spleen and sickle cell disease: the sick(led)spleen. Br J Haematol.

[97] Adamkiewicz TV, Sarnaik S, Buchanan GR, Iyer RV, Miller ST, Pegelow CH, Rogers ZR, Vichinsky E, Elliott J, Facklam RR, O’Brien KL, Schwartz B, Van Beneden CA, Cannon MJ, Eckman JR, Keyserling H, Sullivan K, Wong WY, Wang WC (2003). Invasive pneumococcal infections in children with sickle cell disease in the era of penicillin prophylaxis, antibiotic resistance, and 23-valent pneumococcal polysaccharide vaccination. J Pediatr.

[98] Kizito ME, Mworozi E, Ndugwa C, Serjeant GR (2007). Bacteraemia in homozygous sickle cell disease in Africa: is pneumococcal prophylaxis justified?. Arch Dis Child.

[99] Makani J, Mgaya J, Balandya E, Msami K, Soka D, Cox SE, Komba AN, Rwezaula S, Meda E, Muturi D, Kitundu J, Fegan G, Kirkham FJ, Newton CR, Snow RW, Lowe B (2015). Bacteraemia in sickle cell anaemia is associated with low haemoglobin: a report 5 of 890 admissions to a tertiary hospital in Tanzania. Br J Haematol.

[100] Sakran W, Levin C, Kenes Y, Colodner R, Koren A (2012). Clinical spectrum of serious bacterial infections among splenectomized patients with hemoglobinopathies in Israel: a 37-year follow-up study. Infection.

[101] Al-Salem AH (2011). Splenic complications of sickle cell anemia and the role of splenectomy. ISRN Hematol.

[102] Owusu-Ofori S, Hirst C (2013). Splenectomy versus conservative management for acute sequestration crises in people with sickle cell disease. Cochrane Database SystRev.

[103] Adamkiewicz TV, Silk BJ, Howgate J, Baughman W, Strayhorn G, Sullivan K, Farley MM (2008). Effectiveness of the 7-valent pneumococcal conjugate vaccine inchildren with sickle cell disease in the first decade of life. Pediatrics.

[104] Adamkiewicz TV, Silk BJ, Howgate J, Baughman W, Strayhorn G, Sullivan K, Farley MM (2008). Effectiveness of the 7-valent pneumococcal conjugate vaccine in children with sickle cell disease in the first decade of life. Pediatrics.

[105] Hirst C, Owusu-Ofori S (2014). Prophylactic antibiotics for preventing pneumococcal infection in children with sickle cell disease. Cochrane Database Syst Rev.

[106] Lesher AP, Kalpatthi R, Glenn JB, Jackson SM, Hebra A (2009). Outcome of splenectomy in children younger than 4 years with sickle cell disease. J Pediatr Surg.

[107] Mouttalib S, Rice HE, Snyder D, Levens JS, Reiter A, Soler P, Rothman JA, Thornburg CD (2012). Evaluation of partial and total splenectomy in children with sickle cell disease using an Internet-based registry. Pediatr Blood Cancer.

[108] Afuwape O, Ogole G, Ayandipo O (2013). Splenectomy in a Nigerian Teaching Hospital: A comparison of sonographic correlation with intra-operative findings in trauma. J Emerg Trauma Shock.

[109] Cappellini MD, Cohen A, Porter J, Taher A, Viprakasit V (2014). Guidelines for the Management of Transfusion Dependent Thalassaemia (TDT) [Internet].

[110] Ricerca BM, Di Girolamo A, Rund D (2009). Infections in thalassemia and hemoglobinopathies: focus on therapy-related complications. Mediterr J Hemato Infect Dis.

[111] Merchant RH, Shah AR, Ahmad J, Karnik A, Rai N (2015). Post Splenectomy Outcome in ß-Thalassemia. Indian J Pediatr.

[112] Wang SC, Lin KH, Chern JP, Lu MY, Jou ST, Lin DT, Lin KS (2003). Severe bacterial infection in transfusion-dependent patients with thalassemia major. Clin Infect Dis.

[113] Chirico V, Lacquaniti A, Piraino B, Cutrupi M, Cuppari C, Grasso L, Rigoli L, David A, Arrigo T, Salpietro C (2015). Thalassaemia major and infectious risk: High Mobility Group Box-1 represents a novel diagnostic and prognostic biomarker. Br J Haematol.

[114] Cherchi GB, Pacifico L, Cossellu S (1995). Prospective study of Yersinia enterocolitica infection in thalassemic patients. Pediatr Infect Dis J.

[115] Adamkiewicz TV, Berkovitch M, Krishnan C, Polsinelli C, Kermack D, Olivieri NF (1998). Infection due to Yersinia enterocolitica in a series of patients with b-thalassemia: incidence and predisposing factors. Clin Infect Dis.

[116] Chan GC, Chan S, Ho PL, Ha SY (2009). Effects of chelators (deferoxamine, deferiprone and deferasirox) on the growth of Klebsiella pneumoniae and Aeromonas hydrophila isolated from transfusion-dependent thalassemia patients. Hemoglobin.

[117] Sheikha AK, Salih ZT, Kasnazan KH, Khoshnaw MK, Al-Maliki T, Al-Azraqi TA, Zafer MH (2007). Prevention of overwhelming postsplenectomy infection in thalassemia patients by partial rather than total splenectomy. Can J Surg.

[118] Wong GK, Goldacker S, Winterhalter C, Grimbacher B, Chapel H, Lucas M, Alecsandru D, McEwen D, Quinti I, Martini H, Milito C, Schmidt RE, Ernst D, Espanol T, Vidaller A, Carbone J, Fernandez-Cruz E, Lougaris V, Plebani A, Kutukculer N, Gonzalez-Granado LI, Contreras R, Kiani-Alikhan S, Ibrahim MA, Litzman J, Jones Gaspar HB, Hammarstrom L, Baumann U, Warnatz K, Huissoon AP, Clinical Working Party of the European Society for Immunodeficiencies (ESID) (2013). Outcomes of splenectomy in patients with common variable immunodeficiency (CVID):a survey of 45 patients. Clin Exp Immunol.

[119] Rao VK, Oliveira JB (2011). How I treat autoimmune lymphoproliferative syndrome. Blood.

[120] Price S, Shaw PA, Seitz A, Joshi G, Davis J, Niemela JE, Perkins K, Hornung RL, Folio L, Rosenberg PS, Puck JM, Hsu AP, Lo B, Pittaluga S, Jaffe ES, Fleisher TA, Rao VK, Lenardo MJ (2014). Natural history of autoimmune lymphoproliferative syndrome associated with FAS gene mutations. Blood.

[121] Neven B, Bruneau J, Stolzenberg MC, Meyts I, Magerus-Chatinet A, Moens L, Lanzarotti N, Weller S, Amiranoff D, Florkin B, Bader-Meunier B, Leverger G, Ferster A, Chantrain C, Blanche S, Picard C, Molina TJ, Brousse N, Durandy A, Rizzi M, Bossuyt X, Fischer A, Rieux-Laucat F (2014). Defective anti-polysaccharide response and splenic marginal zone disorganization in ALPS patients. Blood.

[122] Andersson A, Enblad G, Gustavsson A, Erlanson M, Hagberg H, Molin D, Tavelin B, Melin B (2011). Long term risk of infections in Hodgkin lymphoma long-term survivors. Br J Haematol.

[123] Lenglet J, Traullé C, Mounier N, Benet C, Munoz-Bongrand N, Amorin S, Noguera ME, Traverse-Glehen A, Ffrench M, Baseggio L, Felman P, Callet-Bauchu E, Brice P, Berger F, Salles G, Brière J, Coiffier B, Thieblemont C (2014). Long-term follow-up analysis of 100 patients with splenic marginal zone lymphoma treated with splenectomy as first-line treatment. Leuk Lymphoma.

[124] Xing KH, Kahlon A, Skinnider BF, Connors JM, Gascoyne RD, Sehn LH, Savage KJ, Slack GW, Shenkier TN, Klasa R, Gerrie AS, Villa D (2015). Outcomes in splenic marginal zone lymphoma: analysis of 107 patients treated in British Columbia. Br J Haematol.

[125] Rialon KL, Speicher PJ, Ceppa EP, Rendell VR, Vaslef SN, Beaven A, Tyler DS, Blazer DG (2015). Outcomes following splenectomy in patients with myeloid neoplasms. J Surg Oncol.

[126] Barosi G, Ambrosetti A, Buratti A, Finelli C, Liberato NL, Quaglini S, Ricetti MM, Visani G, Tura S, Ascari E (1993). Splenectomy for patients with myelofibrosis with myeloid metaplasia: pretreatment variables and outcome prediction. Leukemia.

[127] Tefferi A, Mesa RA, Nagorney DM, Schroeder G, Silverstein MN (2000). Splenectomy inmyelofibrosis with myeloid metaplasia: a single-institution experience with 22, patients. Blood.

[128] Santos FP, Tam CS, Kantarjian H, Cortes J, Thomas D, Pollock R, Verstovsek S (2014). Splenectomy in patients with myeloproliferative neoplasms efficacy, complications and impact on survival and transformation. Leuk Lymphoma.

[129] Centers for Disease Control and Prevention (CDC) (2012). Use of 13-valentpneumococcal conjugate vaccine and 23-valent pneumococcal polysaccharide vaccine for adults with immunocompromising conditions: recommendations of the Advisory Committee on Immunization Practices (ACIP). MMWR Morb Mortal Wkly Rep.

[130] Mourtzoukou EG, Pappas G, Peppas G, Falagas ME (2008). Vaccination of asplenic or hyposplenic adults. Br J Surg.

[131] Wang J, Jones P, Cheng AC, Leder K (2014). Adherence to infection prevention measures in a statewide spleen registry. Med J Aust.

[132] Serio B, Pezzullo L, Giudice V, Fontana R, Annunziata S, Ferrara I, Rosamilio R, De Luca C, Rocco M, Montuori N, Selleri C (2013). OPSI threat in hematological patients. Transl Med UniSa.

[133] Shapiro ED, Berg AT, Austrian R, Schroeder D, Parcells V, Margolis A (1991). The protective efficacy of polyvalent pneumococcal polysaccharide vaccine. N Engl J Med.

[134] Cherif H, Landgren O, Konradsen HB, Kalin M, Bjorkholm M (2006). Poor antibody response to pneumococcal polysaccharide vaccination suggests increased susceptibility to pneumococcal infection in splenectomized patients with hematological diseases. Vaccine.

[135] Meerveld-Eggink A, de Weerdt O, van Velzen-Blad H, Biesma DH, Rijkers GT (2011). Response to conjugate pneumococcal and Haemophilus influenzae type b vaccines in asplenic patients. Vaccine.

[136] American Academy of Pediatrics (2000). Committee on Infectious Diseases. Policy statement: Recommendations for the prevention of pneumococcal infections, including the use of pneumococcal conjugate, pneumococcal polysaccharide vaccine, and antibiotic prophylaxis. Pediatrics.

[137] Forstner C, Plefka S, Tobudic S, Winkler HM, Burgmann K, Burgmann H (2012). Effectiveness and immunogenicity of pneumococcal vaccination in splenectomized and functionally asplenic patients. Vaccine.

[138] Wu DB, Chaiyakunapruk N, Chong HY, Beutels P (2015). Choosing between 7-, 10- and 13-valent pneumococcal conjugate vaccines in childhood: a review of economic evaluations (2006–2014). Vaccine.

[139] Nived P, Jørgensen CS, Settergren B (2015). Vaccination status and immune response to 13-valent pneumococcal conjugate vaccine in asplenic individuals. Vaccine.

[140] Centers for Disease Control and Prevention (CDC), Advisory Committeeon Immunization Practices (2010). Updated recommendations for prevention of invasive pneumococcal disease among adults using the 23-valent pneu-mococcal polysaccharide vaccine (PPSV23). MMWR Morb Mortal Wkly Rep.

[141] Davies JM, Lewis MP, Wimperis J, Rafi I, Ladhani S, Bolton-Maggs PH, British Committee for Standards in Haematology (2011). Review of guidelines for the prevention and treatment of infection in patients with an absent or dysfunctional spleen: prepared on behalf of the British Committee for Standards in Haematology by aworking party of the Haemato-Oncology task force. Br J Haematol.

[142] Spelman D, Buttery J, Daley A, Isaacs D, Jennens I, Kakakios A, Lawrence R, Roberts S, Torda A, Watson DA, Woolley I, Anderson T, Street A, Australasian Society for Infectious Diseases (2008). Guidelines for the prevention of sepsis inasplenic and hyposplenic patients. Intern Med J.

[143] Salvadori MI, Price VE, Canadian Paediatric Society, Infectious Diseases and Immunization Committee (2014). Preventing and treating infections in children with asplenia or hyposplenia. Paediatr Child Health.

[144] Greenberg RN, Gurtman A, Frenck RW (2014). Sequential administration of 13-valent pneumococcal conjugate vaccine and 23-valent pneumococcal polysaccharide vaccine in pneumococcal vaccinena. ve adults 60–64 years of age. Vaccine.

[145] Jackson LA, Gurtman A, Rice K (2013). Immunogenicity and safety of a 13-valent pneumococcal conjugate vaccine in adults 70 years of age and older previously vaccinated with 23-valent pneumococcal polysaccharide vaccine. Vaccine.

[146] Bonten MJ, Huijts SM, Bolkenbaas M, Webber C, Patterson S, Gault S, van Werkhoven CH, van Deursen AM, Sanders EA, Verheij TJ, Patton M, McDonough A, Moradoghli-Haftvani A, Smith H, Mellelieu T, Pride MW, Crowther G, Schmoele-Thoma B, Scott DA, Jansen KU, Lobatto R, Oosterman B, Visser N, Caspers E, Smorenburg A, Emini EA, Gruber WC, Grobbee DE (2015). Polysaccharide conjugate vaccine against pneumococcal pneumonia in adults. N Engl J Med.

[147] Smith MJ (2008). Meningococcal tetravalent conjugate vaccine. Expert Opin Biol Ther.

[148] O’Ryan M, Stoddard J, Toneatto D, Wassil J, Dull PM (2014). A multi-component meningococcal serogroup B vaccine (4CMenB): the clinical development program. Drugs.

[149] Dotel R, Gosbell IB, Hofmeyr A (2015). Compliance with Australian splenectomy guidelines in patients undergoing post-traumatic splenectomy at a tertiary centre. Med J Aust.

[150] Meerveld-Eggink A, de Weerdt O, van Velzen-Blad H, Biesma DH, Rijkers GT (2011). Response to conjugate pneumococcal and Haemophilus influenzae type b vaccines in asplenic patients. Vaccine.

[151] Meerveld-Eggink A, de Weerdt O, de Voer RM, Berbers GA, van Velzen-Blad H, Vlaminckx BJ, Biesma DH, Rijkers GT (2011). Impaired antibody response to conjugated meningococcal serogroup C vaccine in asplenic patients. Eur J Clin Microbiol Infect Dis.

[152] McCavit TL, Xuan L, Zhang S, Flores G, Quinn CT (2012). Hospitalization for invasive pneumococcal disease in a national sample of children with sickle cell disease before and after PCV7 licensure. Pediatr Blood Cancer.

